# Fractionated irradiation of MCF7 breast cancer cells rewires a gene regulatory circuit towards a treatment‐resistant stemness phenotype

**DOI:** 10.1002/1878-0261.13226

**Published:** 2022-06-15

**Authors:** Auchi Inalegwu, Bart Cuypers, Jürgen Claesen, Ann Janssen, Amelie Coolkens, Sarah Baatout, Kris Laukens, Winnok H. De Vos, Roel Quintens

**Affiliations:** ^1^ Radiobiology Unit, SCK CEN Belgian Nuclear Research Centre Mol Belgium; ^2^ Department of Veterinary Sciences, Faculty of Pharmaceutical, Biomedical and Veterinary Sciences University of Antwerp Belgium; ^3^ Adrem Data Lab, Department of Computer Science University of Antwerp Belgium; ^4^ Department of Biomedical Sciences, Faculty of Pharmaceutical, Biomedical and Veterinary Sciences University of Antwerp Belgium; ^5^ Department of Epidemiology and Data Science, Amsterdam UMC VU Amsterdam The Netherlands; ^6^ Antwerp Centre for Advanced Microscopy (ACAM) University of Antwerp Belgium; ^7^ μNEURO Research Centre of Excellence University of Antwerp Belgium

**Keywords:** breast cancer, circular RNA, radioresistance, relapse‐free survival, stemness, tamoxifen resistance

## Abstract

Radiotherapy is the standard of care for breast cancer. However, surviving radioresistant cells can repopulate following treatment and provoke relapse. Better understanding of the molecular mechanisms of radiation resistance may help to improve treatment of radioresistant tumours. To emulate radiation therapy at the cellular level, we exposed MCF7 breast cancer cells to daily radiation doses of 2 Gy up to an accumulated dose of 20 Gy. Fractionally irradiated cells (FIR20) displayed increased clonogenic survival and population doubling time as compared with age‐matched sham‐irradiated cells and untreated parental MCF7 cells. RNA‐sequencing revealed a core signature of 229 mRNAs and 7 circular RNAs of which the expression was significantly altered in FIR20 cells. Dysregulation of several top genes was mirrored at the protein level. The FIR20 cell transcriptome overlapped significantly with canonical radiation response signatures and demonstrated a remarkable commonality with radiation and endocrine therapy resistance expression profiles, suggesting crosstalk between both acquired resistance pathways, as indicated by reduced sensitivity to tamoxifen cytotoxicity of FIR20 cells. Using predictive analyses and functional enrichment, we identified a gene‐regulatory network that promotes stemness and inflammatory signalling in FIR20 cells. We propose that these phenotypic traits render breast cancer cells more radioresistant but may at the same time serve as potential targets for combination therapies.

## Graphical abstract

Fractionated irradiation induces a treatment‐resistant stemness phenotype underpinned by interaction between competing endogenous RNAs with both tumor‐promoting and tumor‐suppressing function. Results support cross‐resistance of radioresistant MCF7 breast cancer cells to tamoxifen and presents a gene regulatory circuit associated with treatment resistance and survival in breast cancer patients, that could be evaluated for the discovery of novel targeted therapeutic strategies.

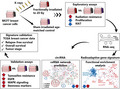

**Abbreviations**
AMC: age‐matched control
ATCC: American type cell culture collection
ceRNA: competitive endogenous RNA
circRNA: circular RNA
CSP: context score percentile
DEC: differentially expressed circRNA
DEM: differentially expressed mRNA
Dt: doubling time
ER+: oestrogen receptor‐positive
FIR20: fractionally irradiated to 20 Gy
GSEA: Gene set enrichment analysis
miRNA: microRNA
mRNA: messenger RNA
MSigDB: Molecular Signatures Database
PAR: parental
PBS: phosphate‐buffered saline
PCA: Principal component analysis
RFS: relapse‐free survival
RNA‐seq: RNA‐sequencing
rRNA: ribosomal RNA
RT: Radiotherapy
rt: room temperature
SF2: surviving fraction at 2 Gy
STRING: Search Tool for the Retrieval of Interacting Genes/Proteins
TBST: tris‐buffered saline with 0.1% Tween
TCGA: The Cancer Genome Atlas

## 1 Introduction

Female breast cancer is the most common cancer diagnosed worldwide [1]. It is also the leading cause of cancer deaths in women with region‐specific mortality rates of 9.8–18.9 per 100 000 women [1]. There were 2.3 million new cases diagnosed in 2020, representing 11.7% of all cancer cases. Radiotherapy (RT) is the standard of care for early breast cancer and is recommended in 83% of all breast cancer patients [2]. It is administered after breast‐conserving surgery or a mastectomy to eliminate residual tumour cells. As such, RT reduces the relapse rate significantly (by a factor of 2/3 to 3) compared to surgery alone [3, 4, 5]. Furthermore, RT combined with adjuvant chemotherapy increases disease‐free survival in patients with high‐risk breast cancer [6, 7]. Nevertheless, tumour recurrence is a major clinical challenge that can occur in 4.3–7.3% of breast cancer patients who receive radiotherapy after breast conserving surgery [8]. Many factors such as total dose, fractionation, tumour doubling time, hypoxia and intrinsic radiosensitivity can impact on the response of tumours to irradiation [9]. Tumour heterogeneity accounts for the varying sensitivity and unequal response of tumour cells to RT. The presence of self‐renewing cancer stem cells is a major contributor to tumour recurrence as well [10]. It has been shown that cancer cells or normal tumour cells can acquire stem cell characteristics that allow them to repopulate after radiation treatment [11, 12, 13]. Studying the population of surviving cells following irradiation can further illuminate the molecular mechanisms of radioresistance that render such tumour cells resilient to the cytotoxic effects of RT [14] and provide insight into developing more precise therapies. In this context, the investigation of gene expression and gene regulatory mechanisms involving both protein coding messenger RNA (mRNA) and noncoding RNA such as microRNA (miRNA) and circular RNA (circRNA) is rapidly gaining significance [15, 16]. Typically, miRNAs negatively regulate gene expression by binding to and mediating the degradation of target mRNA [17], while circRNAs can act as sponges for miRNAs thereby attenuating their effect on mRNA expression [18, 19]. Consequently, different RNA species can cooperate with or antagonize each other within a so‐called competitive endogenous RNA (ceRNA) network [20] to control diverse biological processes and pathways. There is accumulating evidence that circRNAs regulate tumour initiation, pathogenesis and treatment resistance processes by acting as miRNA sponges [21, 22]. Furthermore, circRNA‐associated ceRNA networks have been implicated in chemoresistance, radioresistance, regulation of cancer stem cell properties and populations, evasion of apoptosis, proliferation, migration, and invasion, among other tumour promoting effects [21, 22].

To uncover the transcriptional rewiring that takes place upon radiotherapy, we here exposed oestrogen receptor‐positive (ER+) MCF7 breast cancer cells to fractionated doses of X‐rays. The MCF7 cell line was selected because it is of the hormone receptor‐positive subtype, which accounts for the highest number of breast cancer diagnoses (~ 70%) and shows the best response to RT [23, 24, 25, 26, 27]. Additionally, the MCF7 human breast cancer model has proven to be a reliable preclinical experimental model [28] as it retains several essential features of the mammary epithelium [29] and it is increasingly been used in the search for biomarkers of radiation sensitivity [30, 31, 32, 33]. We used global differential expression profiling, target prediction and functional enrichment approaches to gain insight into the underlying regulatory ceRNA network and its associated biological functions. We found that activation of a cancer stem cell‐like, pro‐inflammatory immune response and several cancer pro‐survival signaling pathways is involved in treatment‐induced radioresistance in breast cancer cells. We also discovered a marked down‐regulation of oestrogen signaling, which likely promotes cross‐resistance to tamoxifen and endocrine therapies.

## 2 Materials and methods

### 2.1 Cell line and cell culture

The human breast cancer cell line MCF7 was purchased from the American type cell culture collection (ATCC LGC Standards, Molsheim Cedex, France). The cells were maintained in Eagle's Minimum Essential Medium (EMEM, ATCC, Middlesex, UK) with 10% Fetal Bovine Serum (Gibco, Life Technologies, Ghent, Belgium) at 37 °C in a humidified incubator with 5% CO_2_ and 95% air. The cells were passaged two times weekly to maintain exponential growth. A cryo‐stock was generated and stored in liquid nitrogen. All experiments were performed using cells thawed from the stock.

### 2.2 Cell lines and irradiation procedure

**Fig. 1:**
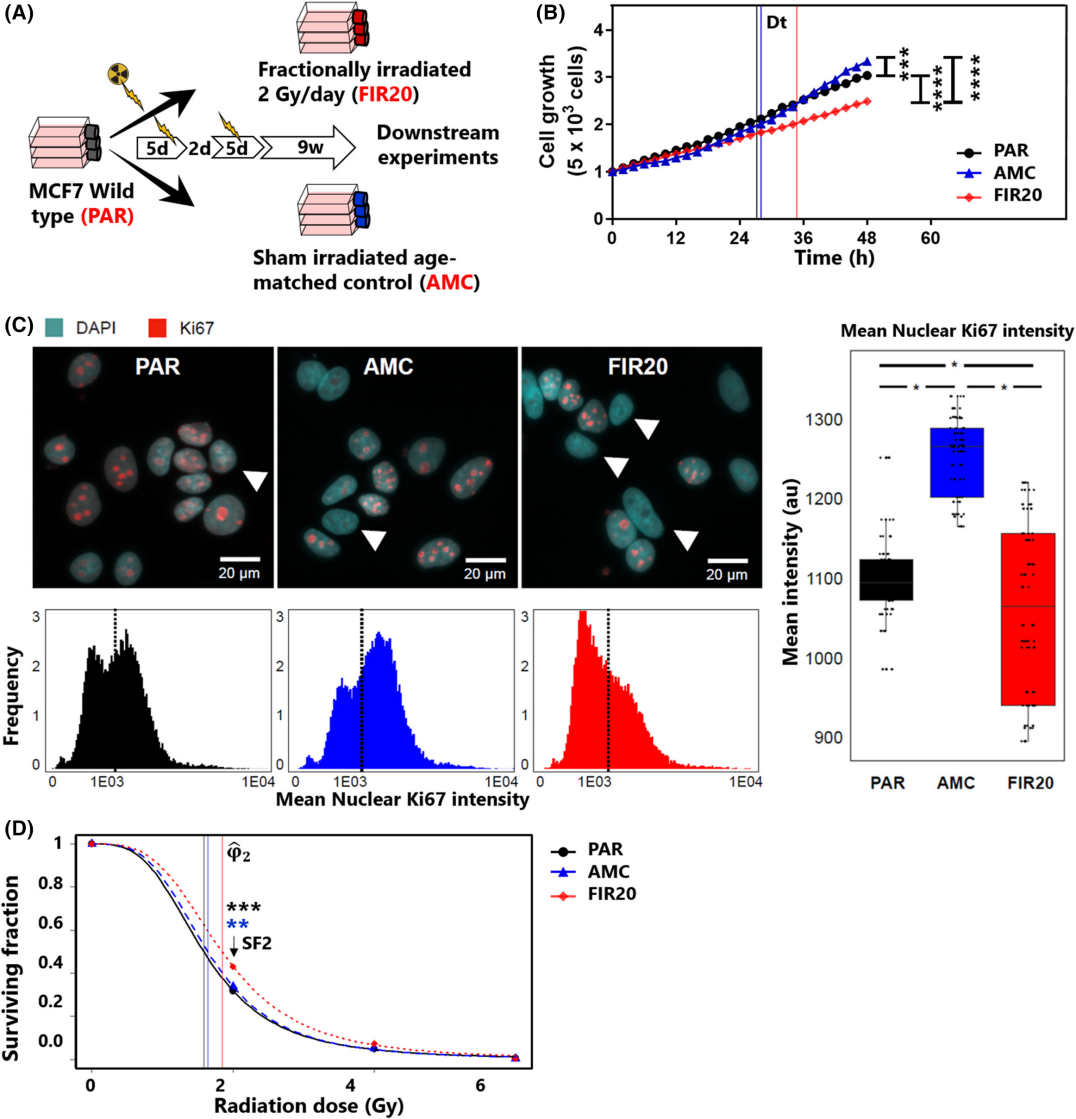
Fractionally irradiated MCF7 cell subline exhibits higher clonogenic survival and doubling time. (A) Overview of fractionated irradiation schedule. The fractionally irradiated (FIR20) cells were obtained by exposing parental (PAR) MCF7 cells to 20 Gy of X‐rays, distributed over a 12‐day period with two series of 5 consecutive days 2 Gy exposure and 2 days without exposure. The age‐matched control group (AMC) group were subjected to the same culture conditions and experimental procedures but were not irradiated. After 2 weeks of irradiation or mock treatment, the cells were allowed to recover for 9 weeks prior to being used for experiments (*n* = 3 biological replicates, d: Days, w: Weeks). (B) Cell proliferation from 0 to 48 h, significance ***P ≤ 0.001, ****P = 0.0001, two‐way ANOVA with holm‐Sidak's multiple comparisons test, *n* = 3. Vertical lines represent the cell doubling time, Dt. (C) Quantitative immunocytochemistry reveals a significantly lower average nuclear Ki67 intensity in FIR20 cells *vs*. PAR and AMC. Note the presence of Ki67 negative cells in all three cell types (arrowheads) (individual image contrast has been adapted for clarity). The box plots show the distribution of the data for each cell line. The boxes indicate the median and interquartile range and whiskers indicate the spread of the data. Statistical analysis was performed using a mixed model with Tukey *post‐hoc* test, *P < 0.05, *n* = 3. Scale bars: 20 μm. (D) Graph of clonogenic assay showing surviving fraction in FIR20, PAR and AMC cell lines after acute exposure to X‐rays at different doses. Stars (*) denote significant differences in surviving fraction following irradiation with 2 Gy. (*) color: Black = FIR20 *vs*. PAR, blue = FIR20 *vs*. AMC, **P = 0.0019, ***P = 0.0001, 2‐way ANOVA with holm‐Sidak's multiple comparisons test, *n* = 3. Vertical lines represent the inflection point ${\hat{\varphi }}_{2}$ of the survival curves . Line colors: Black = PAR, blue = AMC, red = FIR. *N* = 3 for all experiments. [Colour figure can be viewed at wileyonlinelibrary.com]

The sub‐clones used in this study were generated from MCF7 wild type cells referred to here as parental (PAR). PAR cells were grown in three separate T75 cell culture flasks, and each flask of cells was sub‐cultured into 2 sets of 3 cell culture flasks among which one set was assigned to the fractionally irradiated group (FIR20) and the other set was assigned to the age‐matched control group (AMC). The FIR20 group was exposed to 2 Gy fractionated daily doses of X‐rays 5 days per week to a total dose of 20 Gy, and the AMC group were subjected to the same culture conditions and experimental procedures (*e.g*. transport) but were not irradiated, while the PAR cells were maintained in liquid nitrogen storage and only cultured again when needed for downstream experiments (Fig. 1A). Irradiations were performed at a dose rate of 0.5 Gy·min^−1^ using a Xstrahl 320 Kv generator (250 kV, 12 mA, 3.8 mm AI equivalent, 1.4 mm Cu‐filtered X‐rays). To ensure homogeneity, the beam profile was scanned in 2 dimension and measured with a small ionization chamber and cell culture flasks were placed horizontally on the irradiation platform perpendicular to and within the measured range of the X‐ray beam. Cells were subsequently returned to the incubator. Growth medium was changed every 2–3 days, and cells were passaged when they reached 70–90% confluence. The sub‐clones were each generated and maintained in three separate independent cell culture flasks, respectively, throughout the fractionated irradiation scheme and throughout all downstream experiments.

### 2.3 Clonogenic survival assay

Cell survival after single dose irradiation was evaluated using a clonogenic survival assay [34]. MCF7 cell lines in exponential growth phase were harvested by trypsinisation and seeded in triplicate for each radiation dose in 6‐well plates at a density of 0.8 × 10^3^–1 × 10^4^ cells per well, then incubated overnight before irradiation with 0, 2, 4 and 6 Gy, respectively. The cells were maintained for 14–21 days under normal culture conditions as previously described. Cell colonies were fixed with 6% glutaraldehyde and stained with 0.5% crystal violet (Sigma‐Aldrich, Diegem, Belgium). Visible colonies were counted manually and the surviving fraction (SF) was calculated using the formula: number of colonies formed after irradiation/(number of cells seeded x plating efficiency). The surviving fraction (SF(dose)) was estimated using the log‐logistic regression model [35]:

$$\mathrm{SF}\left(\text{dose}\right)=\varphi_{1}+\frac{1-\varphi_{1}}{{\left(1+\exp\left\{\varphi_{3}\left[\ln\left(\text{dose}\right)-\ln\left(\varphi_{2}\right)\right]\right\}\right)}^{\varphi_{4}}}$$

with $\varphi_{1}=0$, $\varphi_{3}$ the parameter proportional to the slope, $\varphi_{2}$ the inflection point of the estimated SF‐curve. The irradiation dose which resulted in 50% survival, also known as the median effective dose (ED50) was represented as ${\hat{\varphi }}_{2}$. The α/β ratio and D10 (radiation dose required to inactivate 90% of cells or reduce clonogenic survival by 90%) were also determined using the linear‐quadratic model [36]. Where β and β parameters describe the cellular radiosensitivity and reflect the linear and quadratic components of cell killing, respectively. α/β = dose at which α and β contribute equally to the total effect.

### 2.4 Cell proliferation and cytotoxicity assays

Real‐time proliferation of cells was assessed using the incucyte zoom (Essen Bioscience, Newark, UK) live‐cell imaging system. 5 × 10^3^ Cells were seeded in 96‐well plates and incubated overnight. Phase‐contrast images were captured every 2 h for 48 h with a 10× objective and analyzed using the integrated incucyte zoom software. Cell proliferation was determined by masking and measuring the percentage surface area occupied by cells (% confluence) which was then expressed in arbitrary units by setting the first time point to 1 for each cell line.

Cytotoxicity assay was used to monitor the effect of tamoxifen on cultured cells using live imaging in a incucyte zoom system (Essen Bioscience). Cytotox green live cell analysis reagent (Essen Bioscience) was used to fluorescently label the nuclei of dying cells after loss of plasma membrane integrity. Briefly, 5 × 10^3^ cells were seeded in 96‐well plates and incubated for 24 h. Cytotox green reagent (250 nm) and 4‐Hydroxytamoxifen (H7904 Sigma‐Aldrich) (0, 1.25, 2.5 or 5 μm) were added to the cells which were then returned to the IncuCyte. Phase‐contrast and fluorescent images were captured every 2 h for 96 h from time of seeding using a 10× objective. Cytotoxicity was measured by counting the number of Cytotox positive nuclei (green object count·mm^−2^) and normalizing to cell confluence over time.

### 2.5 Samples and RNA isolation

Three independent replicates each of exponentially growing PAR, AMC and FIR20 cells were seeded in 25‐cm flasks and incubated for 72 h followed by RNA extraction. Cell pellets were collected by trypsinization, snap frozen and stored at −80 °C until further use. Total RNA was extracted from the cell pellets using RNeasy Mini Kit (Qiagen, Venlo, The Netherlands) as described by the manufacturer. During the extraction procedure, genomic DNA was removed using a genomic DNA eliminator spin column (Qiagen). Sample RNA concentrations were measured and assessed for the presence of contaminants using a NanoDrop ND‐2000 spectrophotometer (Isogen Life Sciences, PW De Meern, The Netherlands) and the DropSense™ 16 spectrometer (Trinean, Ghent, Belgium). RNA integrity was subsequently assessed using Agilent 2100 bioanalyzer (Agilent Technologies, Inc, Palo Alto, CA, USA).

### 2.6 Library preparation and RNA sequencing

High‐throughput whole transcriptome RNA‐sequencing (RNA‐seq) was performed by CD Genomics (Shirley, NY, USA). First, the total RNA was depleted of ribosomal RNA (rRNA) using the Ribo‐Zero HMR kit (Illumina, San Diego, CA, USA) according to the manufacturers instructions. The rRNA‐depleted RNA was then purified using 2x RNAClean XP beads (Beckman Coulter, Brea, CA, USA) followed by first and second‐strand synthesis using NEBNext, New England Biolabs reagents. The generated cDNA was purified with 1.8x SPRIselect Beads (Beckman Coulter). Endprep reaction was performed following the manufacturer's protocol for NEBNext Ultra II End Prep Reaction for the rest of the procedure. Adaptor ligation was then performed using NEBNext Ultra II Ligation protocols (New England Biolabs, Ipswich, MA, USA), and the ligated product was purified by SPRIselect Beads (Beckman Coulter) followed by elution in nuclease‐free water. PCR was carried out using NEBNext Ultra II Q5 Master Mix, and primers and the final library was subsequently purified with SPRIselect Beads. Libraries were sequenced using the Illumina HiSeq platform, generating 2 × 150 bp paired‐end reads and at least 50 million reads per library according to the manufacturer's instructions.

### 2.7 Bioinformatics

The quality of the paired‐end reads was verified using fastqc version 0.11.8 [37]. Reads were mapped to the Human genome assembly GRCh38.p13 (Genome Reference Consortium Human genome build 38 – with Gencode) and count tables generated using star v2.7.3a [38] for mRNA detection. During this first run of STAR, the option ‘chimSegmentMin’ was set to 10 to enable the detection of chimeric alignments. Subsequently, a ‘second pass’ run of STAR was carried out with the option ‘sjdbFileChrStartEnd’ for the most sensitive novel junction discovery. Circular RNAs were identified and quantified using the CIRCexplorer2 [39] and CIRI2 [40] tools and subsequently annotated from the circBase database [41]. DESeq2 [42] was used for data normalization and to screen differentially expressed mRNA (DEM) and circRNA (DEC). Significant differential expression was defined as those DEMs having an absolute log2 fold‐change (log2FC) larger than 1 and an adjusted *P*‐value below 0.05. For DECs, a (nonadjusted) *P*‐value ≤ 0.001 was considered significant. Gene set enrichment analysis (GSEA) [43] of the global gene expression data was performed using DESeq2‐normalized transcript counts [42]. A weighted enrichment statistic was used with a signal‐to‐noise metric for gene ranking [43]. Results with FDR <0.25 were considered significant. The ‘investigate gene sets’ online tool of the Molecular Signatures Database (msigdb v7.0) [44] was used for functional enrichment analysis of the DEMs. Prediction and visualization of protein–protein interaction networks were performed using the Search Tool for the Retrieval of Interacting Genes/Proteins (STRING) database online resource [45].

CircInteractome [46], and miRWalk [47] online databases were used to predict the circRNA target miRNAs and their respective target mRNAs. We first predicted miRNAs that could be bound/sponged by DECs using CircInteractome. Then, mRNA targets of the identified miRNAs were predicted using miRWalk's ‘validated miRNA targets module’. The DECs and their predicted targets were used to construct a competitive endogenous RNAs (ceRNA) network of circRNA‐miRNA‐mRNA regulatory interactions. Intersection(s) of gene lists were calculated using the Venn diagram Web tool at https://bioinformatics.psb.ugent.be/webtools/Venn/. Only genes predicted by mirWalk that were also DEMs were used to construct and visualize the network using cytoscape software (Version 3.8.2) [48]. CircInteractome uses the TargetScan algorithm to predict miRNA‐circRNA interactions by searching for 7‐mer or 8‐mer complementarity to the seed region and the 3′ end of the miRNAs [49]. We filtered for circRNA‐miRNA interacting pairs with a context score percentile (CSP) > 90 to select for high‐confidence miRNAs targets with high binding specificity. Parameters for target scoring based on CSP are detailed in Grimson *et al* [50]. The context of effective sites is scored for favorable binding sites. The CSP is the percentile rank of each site compared with all sites for a given miRNA family. Therefore, a high CSP designates a specific site as being more favorable for binding than most other sites for a miRNA.

### 2.8 Quantitative real‐time PCR

Quantitative real‐time PCR (qPCR) was used to validate a selection of DEMs and DECs. Total RNA was isolated from the study cell lines (*i.e*., PAR, AMC and FIR20) using RNeasy Mini Kit as previously described. Subsequently, cDNA was synthesized from 1 μg of the extracted total RNA using GoScript™ Reverse Transcription kit (Promega Corporation, Leiden, The Netherlands) and amplified by qPCR based on the SYBR green method on an ABI 7500 Fast Real‐Time PCR System (ThermoFisher Scientific, Ghent, Belgium) or a Rotor‐Gene Q system (Qiagen, Hilden, Germany). The reference genes GAPDH and TFRC were used for normalization. The convergent target‐specific primers used for mRNA detection were designed using NCBI primer‐BLAST tool [51] and divergent primers spanning the circRNA back‐splice junctions were designed using CircInteractome Webtool [46]. All the primer sequences were synthesized by Eurogentec (Seraing, Belgium), and their sequences are listed in Table S1. All experiments were performed with three independent biological replicates of the respective samples and two technical replicates. Relative mRNA and circRNA expression levels were compared using the Pfaffl method [52].

### 2.9 Protein isolation and detection

Protein lysates were prepared by lysing cells in ice‐cold RIPA Lysis and Extraction Buffer (89900, Thermo Scientific, Merelbeke, Belgium). Whole cell lysates were collected and centrifuged at 16 900 *g* for 15 min at 4 °C, and protein concentration was determined using the Bicinchoninic acid Assay. Equal amount of protein was separated by sodium dodecyl sulphate SDS gel electrophoresis and blotted onto polyvinylidene difluoride (PVDF) membranes. The membranes were blocked for 1 h in TBST (tris‐buffered saline with Tween 20, 0.1%) containing 5% bovine serum albumin, then incubated overnight at 4 °C with primary antibodies (Table S2). Afterwards, the membranes were washed 3 times with TBST for 5 min at room temperature (rt) with rocking, and incubated with horseradish peroxidase‐conjugated secondary antibodies (goat‐anti‐mouse‐62‐6520/goat‐anti‐rabbit‐65‐6120 ‐ Invitrogen, Merelbeke, Belgium) for 1 h. Signals were detected by chemiluminescence reagents (Bio‐Rad Laboratories, Temse, Belgium). Images were acquired with Fusion FX. Membranes required for re‐probing with beta‐actin antibody were stripped using Restore™ PLUS Western Blot Stripping Buffer (Thermofisher, https://www.thermofisher.com/order/catalog/product/46430). Semi‐quantitative analysis of intensity of the bands was performed using imagej [53]. Signals in AMC were set at 1.00.

### 2.10 Quantitative immunocytochemistry (ICC) and cell cycle analysis

Paraformaldehyde‐fixed cultures (2%, 30 min, rt) were permeabilized with 0.3% Triton X‐100 for 8 min and incubated in blocking buffer (50% fetal bovine serum in PBS) for 30 min, followed by a 60‐min incubation with primary antibodies (Table S2). After washing with PBS, secondary antibodies (Donkey‐anti‐Mouse‐Cy3/Donkey‐anti‐Rabbit‐Cy5) were added for 30 min at room temperature. Finally, DAPI was applied to the cultures for 15 min at a concentration of 2.5 μg·mL^−1^, followed by a PBS wash. Fluorescent images were collected with an automated Nikon Ti‐E inverted microscope (Nikon Instruments Europe, Amsterdam, The Netherlands) equipped with a SPECTRA light engine® solid‐state light source (Lumencor, Beaverton, USA) and a Nikon DS‐Qi2 digital camera. Nikon NIS‐elements software was used to control the image acquisition. Analysis of immunofluorescently labeled cells was made in fiji software using an updated version of a high content cell profiling script that has been developed earlier [54, 55] (CellBlocks.ijm), and which is available on Github (https://github.com/DeVosLab/CellBlocks). In brief, nuclei were detected in maximum projections of confocal Z‐stacks of the DAPI channel after local contrast enhancement to cover for spatial illumination heterogeneity using StarDist versatile (fluorescent nuclei) neural network model, with a probability score of 0.30 and an overlap score of 0.20. After size filtering, the average intensity of the immunolabeled markers was measured within the nuclear regions of interest. The integrated intensity of the DAPI signal was used as a proxy for DNA content and cell cycle estimation as previously done [56].

### 2.11 Investigation of patient data

To assess the relationship between the gene of interest and outcome in breast cancer patients, we used the Kaplan–Meier plotter at http://kmplot.com/analysis/ for survival analysis. Sources for the the Kaplan–Meier plotter database include GEO, EGA and TCGA. The characteristics of patients in the data set have been described previously [57]. We evaluated relapse‐free survival (RFS) of patients who received endocrine treatment alone (*n* = 867, 84.3% ER+), tamoxifen alone (*n* = 733, 90.3%), chemotherapy (*n* = 844, 19.4% ER+) or systematically untreated patients (*n* = 1025, 48% ER+). The analysis was performed by grouping patient samples based on the expression of the gene of interest using the Jetset best Probeset and the median cut‐off value [57]. Additionally, association between genes of interest and survival status among patients who received radiotherapy was assessed using data from whole‐exome sequencing of 817 breast invasive carcinoma (ER status IHC % positive, 37.5%) tumour/normal pairs [58] from The Cancer Genome Atlas (TCGA) database available in cBioPortal at https://www.cbioportal.org/study/clinicalData?id=brca_tcga_pub2015 [59, 60]. The relationship between gene of interest and outcome was assessed by fitting a basic Cox‐proportional‐hazard model for survival status. Patients were grouped based on the expression‐levels of the gene of interest: ‘above the median expression level of the gene’ (*n* = 409) and ‘below the median expression level of the gene’ (*n* = 408). Furthermore, we evaluated the association of the core signature genes with tumour stage in patients with breast invasive carcinoma from TCGA (*n* = 1085) using Gene Expression Profiling Interactive Analysis (GEPIA) an interactive web tool for analyzing RNA‐sequencing expression data from the TCGA and the Genotype‐Tissue Expression (GTEx) projects (http://gepia.cancer‐pku.cn/) [61]. Ethical approval was not required because all the patient data in this study were accessed and/or downloaded from the publicly available databases Kaplan–Meier plotter, GEPIA and cbioportal. The TCGA data were generated by the TCGA Research Network: https://www.cancer.gov/tcga.

### 2.12 Statistical analysis

All experiments were conducted in triplicate using three independently generated cell lines each with at least three technical replicates for functional assays and two technical replicates for RT‐qPCR. Two‐way ANOVA with Holm‐Sidak's multiple comparison test was used to test for differences between two groups for colony forming and proliferation assays. For qPCR, one‐way ANOVA with Holm‐Sidak's multiple comparison test was used to test for differences between FIR20 cells and the control cells (PAR and AMC). Differences with *P*‐values < 0.05 were considered statistically significant. Data are presented as mean ± 1.96 SEM or SD, and statistical analysis were performed with graphpad prism version 9.2.0 (San Diego, CA, USA). Cytotoxicity assays were analyzed with a linear mixed effects model, after log‐transforming the cytotoxicity values. For ICC, downstream data analysis was performed in r studio [62]. For DAPI‐based cell cycle staging, the integrated intensity values were normalized by dividing with the average intensity per replicate. For other markers, no additional correction was applied. Statistical comparisons were performed using a linear mixed effect model with cell type as fixed factor and the plate (biological replicate) as a random variable with well (technical replicate) as a nested factor.

## 3 Results

### 3.1 Fractionally irradiated MCF7 cells exhibit reduced basal proliferation and increased clonogenic survival after radiation

To assess the impact of fractionated irradiation on cell physiology, we monitored the basal cell proliferation potential using quantitative, label‐free, live cell imaging. We found that the FIR20 cells proliferate slower than PAR and AMC cells as indicated by a significant increase in their doubling time (Dt) (Fig. 1B, Table S3). In line with this, quantitative ICC staining revealed a significant decrease in the average nuclear intensity of the proliferation marker Ki67 in FIR20 cells (Fig. 1C). However, no significant shift in cell cycle profile was detected using DAPI‐based cell cycle staging (Fig. S1).

Next, we examined whether fractionated exposure to radiation would render MCF7 cells more resistant to a subsequent exposure. To this end, a clonogenic survival assay was performed after exposing the PAR, AMC and FIR20 cells to a single dose of X‐irradiation ranging from 0 to 6 Gy. The survival curve of the FIR20 cells was characterized by a significantly higher φ_2_ (ED_50_) and SF2, and a lower α/β ratio. These results suggest a competitive survival advantage of FIR20 cells compared with the controls to a subsequent radiation exposure (Fig. 1D, Table S4).

### 3.2 FIR20 transcriptome exhibits a profile of radioresistance, cross‐resistance to anti‐oestrogen therapies and luminal‐to‐basal subtype plasticity

**Fig. 2:**
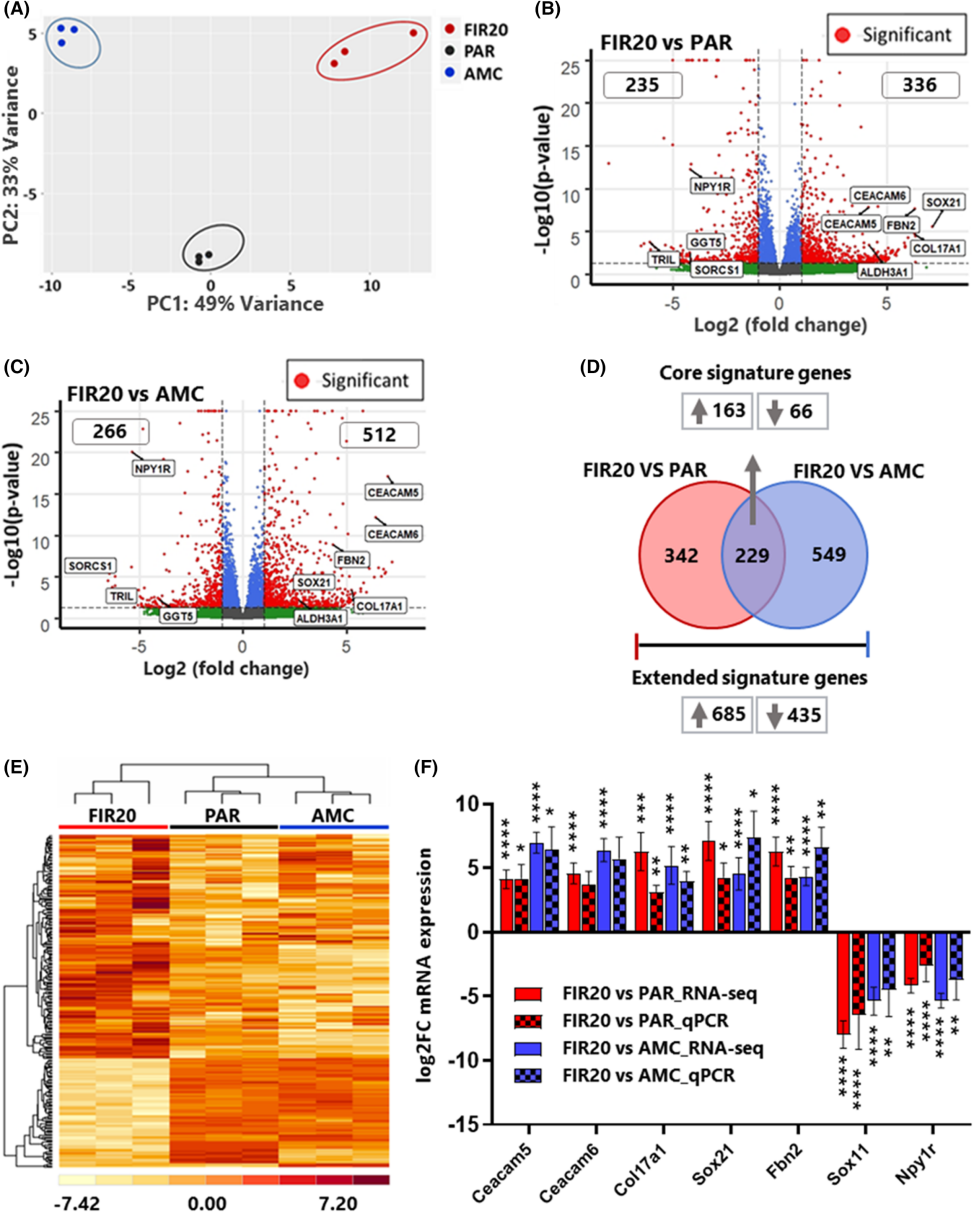
Identification of differentially expressed mRNAs. (A) Biplot of principal component analysis showing the variation of the respective gene sets. Clustering of the gene sets by cell line are specified by the ellipses. (B, C) volcano plots showing the *P* value (*y*‐axis) and the fold change (*x*‐axis) for the differentially expressed genes between FIR20 *vs*. PAR and FIR20 *vs*. AMC respectively. Red dots indicate significantly upregulated and downregulated mRNAs *P*adj ≤ 0.05;¦log_2_FC¦ ≥ 1. Some of the shared DEMs and those validated by qPCR are indicated on the plots. Genes with significance values ≤ 10e‐25 were set to 10e‐25. (D) Venn diagram showing the 229 common DEMs (core signature genes) and 1120 extended signature genes. (E) Heat map showing hierarchical clustering of the core signature genes. (F) Results of qPCR analysis of 7 top DEMs in the core signature presented as mean ± (1.96 SE) of three biological replicates. *P < 0.05; **P < 0.005, ***P = 0.0001, ****P < 0.0001; by one‐way ANOVA with holm‐Sidak's multiple comparisons test. FC, fold change. The RNA‐seq data plotted are based on CIRCexplorer2 results. *N* = 3. [Colour figure can be viewed at wileyonlinelibrary.com]

To investigate the effect of fractionated irradiation on gene expression, we used RNA‐seq to generate mRNA expression profiles of the PAR, AMC and FIR20 cells. Principal component analysis (PCA) of the resulting transcriptomes revealed a clear grouping of the biological replicates of the parental and derivative cell lines proving reproducibility (Fig. 2A). In FIR20 cells, 571 and 778 mRNAs were significantly differentially expressed (DEM, adjusted *P*‐value ≤ 0.05 and log2FC ≥ 1 or ≤ −1) compared with the PAR cells and AMC controls, respectively (Fig. 2B,C and Suppl. Data S1). Among these, 229 DEMs were conserved and demonstrated a concordant pattern of up‐ or down‐regulation (163 up and 66 down, respectively) for both comparisons (*i.e*. FIR20 *vs*. AMC and FIR20 *vs*. PAR, respectively) (Fig. 2D,E). As both FIR20 and AMC cells may have experienced (epi‐) genetic drift from the PAR cell line during sustained culture, we considered this subset of common DEMs as the *core radio‐adaptive signature* in the FIR20 cells (Fig. 2D, Suppl. Data S2) and examined the corresponding genes to further interpret the FIR20 phenotype. For a comprehensive overview, we also examined the total subset of genes altered in FIR20 *vs*. either one of both controls (PAR and AMC cells) including the shared and unique DEMs (Fig. 2D, Suppl. data S3). We named this the *extended radio‐adaptive signature*. A selection of 7 top hits from the core signature was validated using qPCR on samples from independent cell cultures, revealing very similar changes as those detected by RNA‐seq (Fig. 2F).

**Fig. 3:**
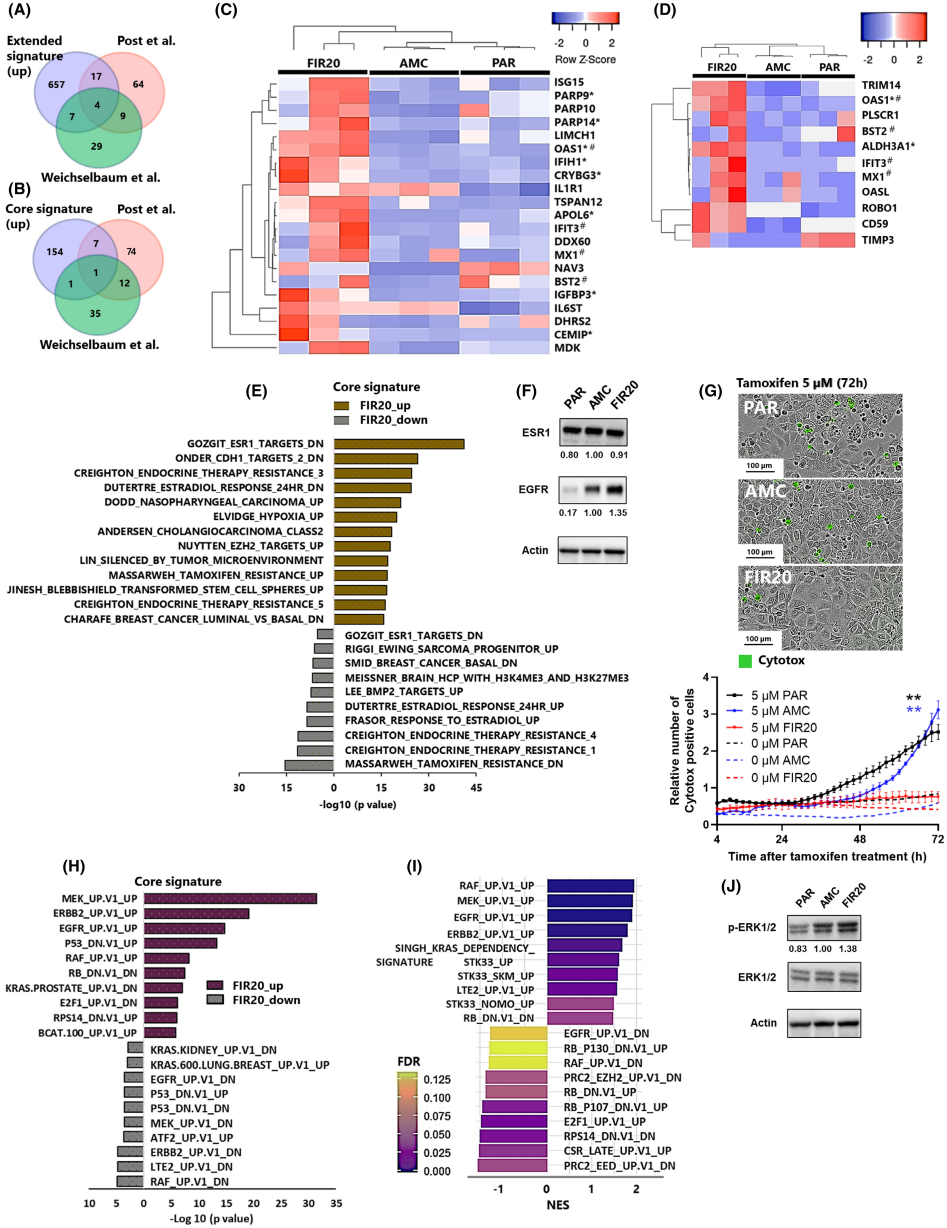
FIR20 cells exhibit a treatment resistant, basal‐like gene expression profile underlined by increased levels of EGFR expression and MAPK pathway. Overlap of genes with increased expression in (A) the extended signature and (B) core signature with Post *et al*. [31] genes which were increased in both tamoxifen and radiation resistant cells and Weichselbaum *et al*. [33] genes which are associated with chemotherapy and/or radiation resistance. Heat map showing the expression of the extended signature genes that overlapped with (C) Post *et al* gene sets and (D) Weichselbaum *et al*. genes, respectively. Red indicates higher expression, blue indicates lower expression. #genes present in both Post *et al*. and Weichselbaum *et al*. signatures, *genes also present in the core radio‐adaptive signature. Clustering was performed using complete linkage (C) and average linkage (D) with Euclidean metric. (E) MSigDB C2: Curated pathways dysregulated in FIR20 cells based on enrichment analysis of the core signature genes showing dysregulation of ESR1 signaling, enrichment of tamoxifen and endocrine therapy resistance gene signatures, and basal‐like breast cancer genes. (F) Western blot analysis showing the expression profiles of ESR1 and EGFR in PAR, AMC and FIR20 cell lines, *n* = 3 (G) images (top) and graph (bottom) showing cytotoxicity of 5 μm tamoxifen in PAR, AMC and FIR20. Images (72 h after tamoxifen treatment), graph (4–72 h) after tamoxifen treatment. Linear mixed effect model with Tukey's honest significant difference test, **P ≤ 0.01 significance between both controls and FIR20, *n* = 3. Data is presented as mean ± (1.96 SE) of three biological replicates. Scale bars: 100 μm. Oncogenic signatures enriched in (H) the core signature and (I) FIR20 *vs*. REST respectively. (J) Western blot analysis confirmed enrichment of MAPK pathway in FIR20 cells, *n* = 3. Bands from western blotting were semi‐quantitatively analyzed using imagej. Signals in AMC were set at 1.00 but no statistical analysis was performed. [Colour figure can be viewed at wileyonlinelibrary.com]

To confirm the observed radioresistant‐like phenotype of the FIR20 cells at the transcriptional level, we compared the genes of the extended and core signatures with increased expression in FIR20 cells with 2 published gene expression signatures associated with radiation resistance [31, 33] (Fig. 3A–D, Table S5). The first [31] contained a list of 94 genes with increased expression in both treatment‐derived radioresistant and tamoxifen‐resistant MCF7 breast cancer cells relative to sensitive controls. The second [33] consisted of an interferon‐regulated signature of 49 genes implicated in radiation and/or chemotherapy resistance. Of the extended signature, 21 genes were present in the Post *et al*. gene set (22%) and 11 in the Weichselbaum *et al*. gene set (22%) (Fig. 3A). Of the core signature, there were 8 (9%) and 2 (4%) genes in common with these gene sets, respectively (Fig. 3B). These results indicate that the gene expression profile of FIR20 cells shows commonality with previously described breast cancer cells with acquired resistance to radiation and cross‐resistance to tamoxifen.

To explore the significant pathways associated with the radioresistant‐like phenotype, we performed functional enrichment analysis of the FIR20 core signature using the MSigDB gene set investigation tool [44] (Data S2). We also used GSEA [43] for exploratory analysis of the global transcriptome of the FIR20 cells compared with PAR and AMC cells (‘*FIR20 vs. REST*’) using the complete DESeq2 normalized reads of all the expressed mRNA transcripts (Fig. S2, Data S4). First, we queried for ‘genetic and chemical perturbations’ within the MSigDB curated gene set. A striking observation was the significant dysregulation in FIR20 cells of oestrogen receptor alpha (ESR1) target genes and gene sets associated with tamoxifen and endocrine therapy resistance (Fig. 3E, Data S2). Western blotting revealed no difference in ESR1 protein levels suggesting that the observed changes are rather related to changes in receptor activation or downstream signaling proteins (Fig. 3F). To assess the impact of altered oestrogen receptor signaling on hormone therapy response, we evaluated the effect of tamoxifen in the cell lines. We found a strong attenuation of tamoxifen‐induced cytotoxicity in FIR20 cells (Fig. 3G, Fig. S3). These findings align with the notion that resistance to anti‐oestrogen and radiation therapy involve common effector genes [31]. We also gathered evidence for additional cross‐resistance, as several genes associated with ERBB2 expression or ERBB2 subtypes of breast cancer and genes involved in the response and resistance to chemotherapeutic agents such as fenretinide, fluorouracil, doxorubicin and the EGFR inhibitor Gefitinib were significantly dysregulated in the FIR20 cells (Fig. S2, Data S4). Western blotting revealed higher EGFR levels in FIR20 cells arguing for a direct link with this receptor (Fig. 3F). In fact, EGFR was part of a cluster of dysregulated genes and enriched gene sets in the FIR20 cells that define the basal breast (cancer) subtype (*LAMC2, TRIM29, COL17A* and cytokeratin genes (*KRT6, KRT7, KRT13, KRT15*)) (Data S1). We therefore conclude that FIR20 cells exhibit an altered molecular landscape that confers resistance to radiation, reduced sensitivity to endocrine therapies and enhanced EGFR signaling.

### 3.3 FIR20 transcriptome exhibits characteristic profile of MAPK pathway activation

Having observed a strong change in EGFR, we next queried the MSigDB C6 collection for oncogenic pathway activation. We found significant overlap between the expression profile of FIR20 cells with gene sets previously reported as being dysregulated in MCF7 breast cancer cell models engineered for hyperactivation or overexpression of the mitogen‐activated protein kinase (MAPK) pathway [63] (Fig. 3H,I, Data S2 and S4). Western blot analysis confirmed increased levels of phosphorylated ERK 1/2 in FIR20 cells compared with PAR and AMC confirming upregulation of the MAPK pathway (Fig. 3J).

### 3.4 FIR20 transcriptome inclines towards cancer cell stemness

**Fig. 4:**
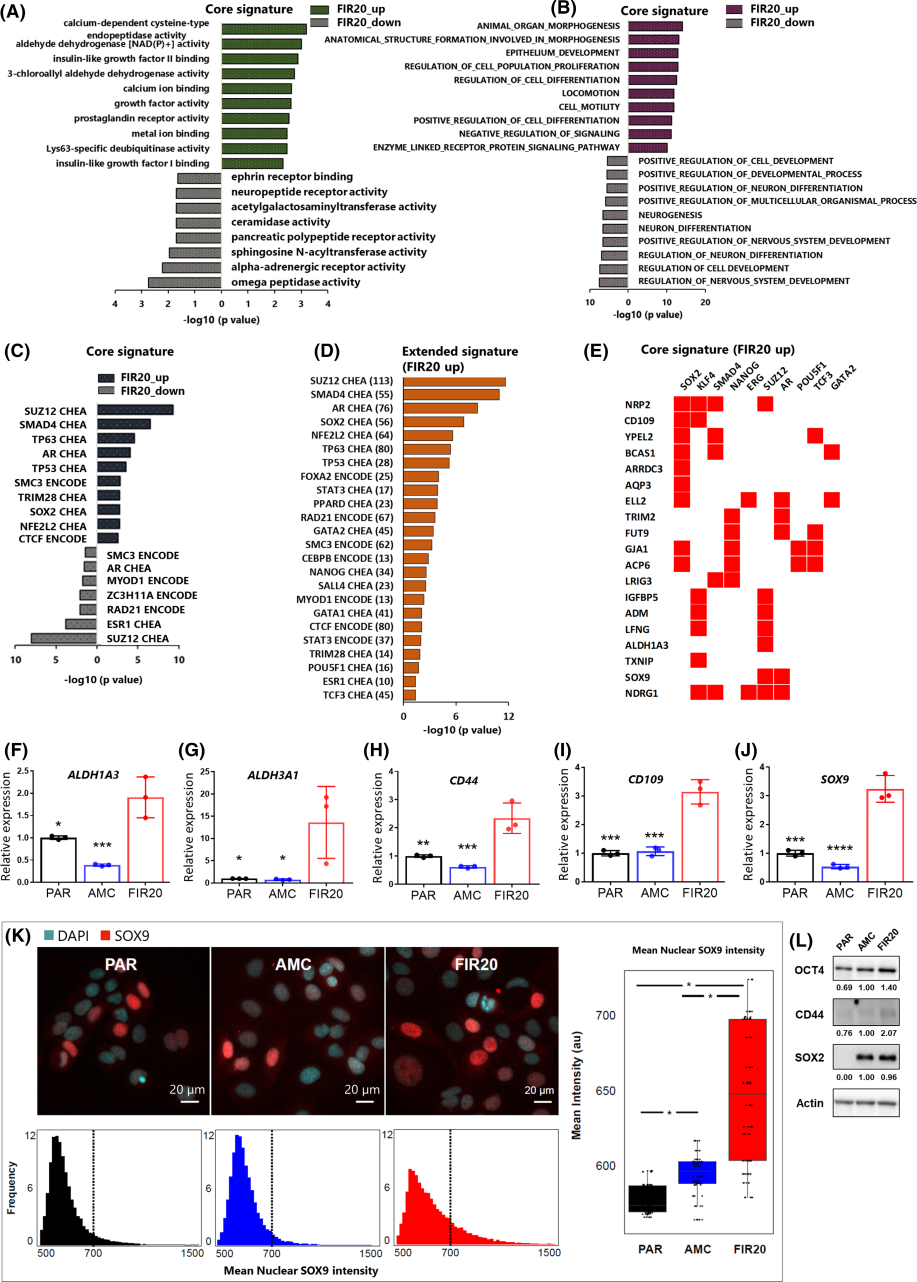
FIR20 cells display a cancer stem cell‐like gene expression profile and induction of genes regulated by pluripotency‐associated transcription factors. Gene ontology analysis of the core signature DEMs for (A) molecular functions and (B) biological processes. Transcription factor enrichment analysis of (C) the core signature genes and (D) extended signature genes performed using Enrichr [112]. Transcription factors were extracted from the ENCODE and ChIP enrichment analysis (ChEA) consensus TFs from ChIP‐X database [112]. (E) Heat map showing core signature genes on the y‐axis and their predicted pluripotency associated TF and other TFs which share the same targets on the *x*‐axis based on hits extracted from (D). Red boxes represent transcription factor‐target gene relationship. (F–J) graphs showing relative expression of stem cell‐associated genes in PAR, AMC and FIR20 cells validated by qPCR. Data is represented as mean ± SD of three biological replicates normalized to expression in wild‐type PAR cells, and statistical significance was determined by one‐way ANOVA with holm‐Sidak's multiple comparison test. mRNA expression, *P < 0.05; **P < 0.005, ***P = 0.0001, ****P < 0.0001, significant differences are relative to FIR20. (K) Immunocytochemistry  staining indicating increased levels of SOX9 in FIR20 cells compared to controls. The box plots show the distribution of the data for each cell line. The boxes indicate the median and interquartile range and the whiskers indicate the spread of the data. Statistical analysis was performed using a mixed model with Tukey *post‐hoc* test, *P < 0.05. Scale bars: 20 μm. (L) Western blot analysis showing increased expression of OCT4 (POU5F1) and CD44 in FIR20 cells. Bands from western blotting were semi‐quantitatively analyzed using imagej. Signals in AMC were set at 1.00. K and L, *n* = 3. [Colour figure can be viewed at wileyonlinelibrary.com]

FIR20 cell transcriptome demonstrated enrichment in MSigDB gene sets associated with blebbishield transformation an emergency program that promotes cell transformation at the onset of apoptosis [64] in cancer stem cells (Fig. 3E, Data S2). Gene ontology analysis provided further evidence of induction of stem cell and pluripotency‐associated genes in the FIR20 cells (Fig. 4). For example, the core signature was enriched in the GO molecular function (Fig. 4A) ‘aldehyde dehydrogenase [NAD(P)+] activity’ attributed to the increased expression of the stemness markers *ALDH1A3*, *ALDH3A1* and several GO biological processes relating to cell differentiation and development attributed to cancer stem cell associated genes such as *CD109* and *SOX9*. (Fig. 4B). Upstream transcription factor prediction (Fig. 4C–E, Data S2 and S3) revealed that many genes of the core signature were targets of pluripotency factors SOX2, POU5F1 (OCT4), NANOG and SALL4. We validated the increased expression of *ALDH1A3, ALDH3A1, CD44, CD109* and *SOX9* in FIR20 cells using qPCR (Fig. 4F‐J), and confirmed upregulation at the protein level for SOX9 using quantitative immunofluorescence (Fig. 4K), as well as OCT4 and CD44 using western blots (Fig. 4L). Despite the enrichment of its transcriptional target genes in FIR20, SOX2 did not change significantly with respect to AMC cells (Fig. 4L). This can be explained by the dependence of transcription factor activity on their shuttling kinetics [65] and/or the presence of binding partners [66] rather than abundance alone. Our results show that the enriched stemness and pluripotency‐associated factors, possibly in crosstalk with the other transcriptional regulators (*SUZ12, SMAD4, AR, TCF3, TP53*), may drive the core radio‐adapted transcriptome of FIR20 cells and portray a stem‐cell like, radiation‐induced cellular reprogramming of this cell line.

### 3.5 Core signature genes are correlated with worse survival and tumour stage

**Fig. 5:**
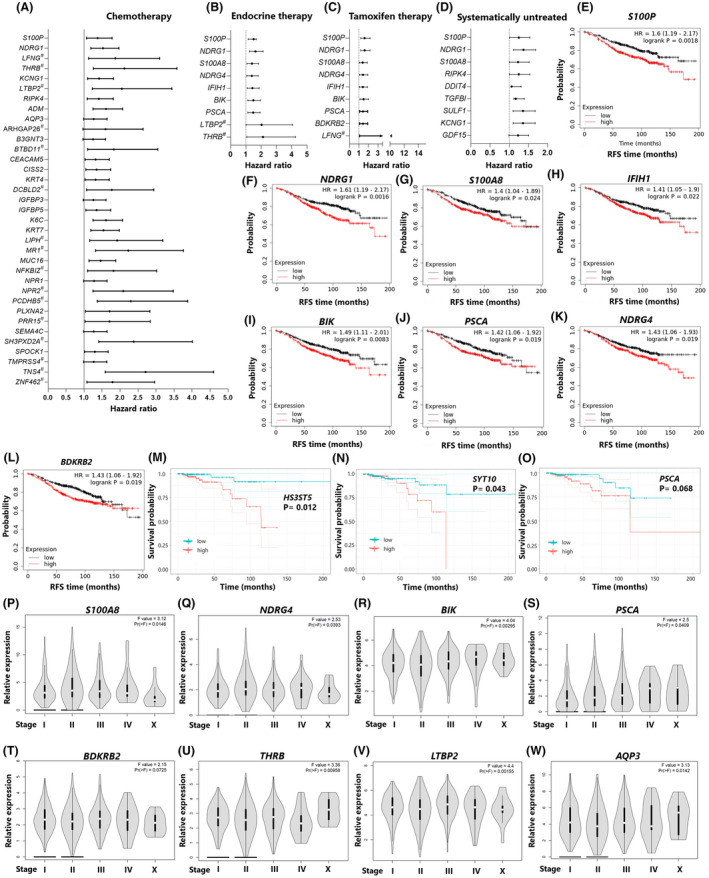
Core signature genes are associated with ‐ poorer survival after chemotherapy, endocrine therapy, tamoxifen therapy or radiotherapy and tumour stage. (A–D) Forest plots showing the association (hazard ratios and 95% CIs) between the expression of the core signature genes and relapse‐free survival in breast cancer patients after chemotherapy (*n* = 844, # *n* = 211), endocrine therapy (*n* = 867, # *n* = 181) or tamoxifen treatment (*n* = 733, # *n* = 110) or no systemic treatment (*n* = 1025) [57]. Error bars represent 95% CI. (E–L) Kaplan–Meier plots of relapse‐free survival in high and low expression groups for the core signature genes which correlated with poor survival in endocrine or tamoxifen treated patients. Not shown: *LFNG*, *LTBP2* and *THRB*. Cox proportional hazard survival analysis, a log‐rank *P* < 0.05 was considered as statistically significant. (M–O) plots of overall survival in high and low expression groups for core signature genes which correlated with poorer overall survival in breast cancer patients treated with radiotherapy based on a cox proportional hazard model, significance level 0.05. *N* = 817. (P–W) violin plots illustrating the expression levels of 8 core signature genes that were associated with tumour stage in breast invasive carcinoma generated from TCGA using GEPIA, *n* = 1085. RFS, relapse‐free survival. [Colour figure can be viewed at wileyonlinelibrary.com]

To test the clinical relevance of our results, we examined the association of the core signature genes with relapse‐free survival in breast cancer patients treated with chemotherapy, endocrine therapy or no systematic treatment, using Kaplan–Meier analysis [57]. The results showed that high expression levels of many core signature genes were negatively correlated with relapse‐free survival in patients who received chemotherapy (35), endocrine therapy (9), tamoxifen (9) or no systematic therapy (9), respectively (Fig. 5A–L, Data S5). High expression of two genes were also associated with worse overall survival in breast cancer patients treated with radiotherapy based on analysis of TCGA data [58] available on cBioPortal (Fig. 5M–O). The effect of the core signature genes on the pathogenesis of breast cancer was investigated using GEPIA [61] to determine their association with tumour stage based on analysis of samples from the TCGA project (Data S5). Six genes which were inversely correlated with relapse‐free survival in endocrine and/or tamoxifen‐treated patients also correlated with tumour stage (Fig. 5P–W). Six other genes from the chemotherapy and/or systematically untreated group also significantly correlated with tumour stage in breast cancer patients. Thus, we conclude that radioresistant cancer cells are associated with a worse prognosis.

### 3.6 Fractionated irradiation modulates the expression of circular RNAs

**Fig. 6:**
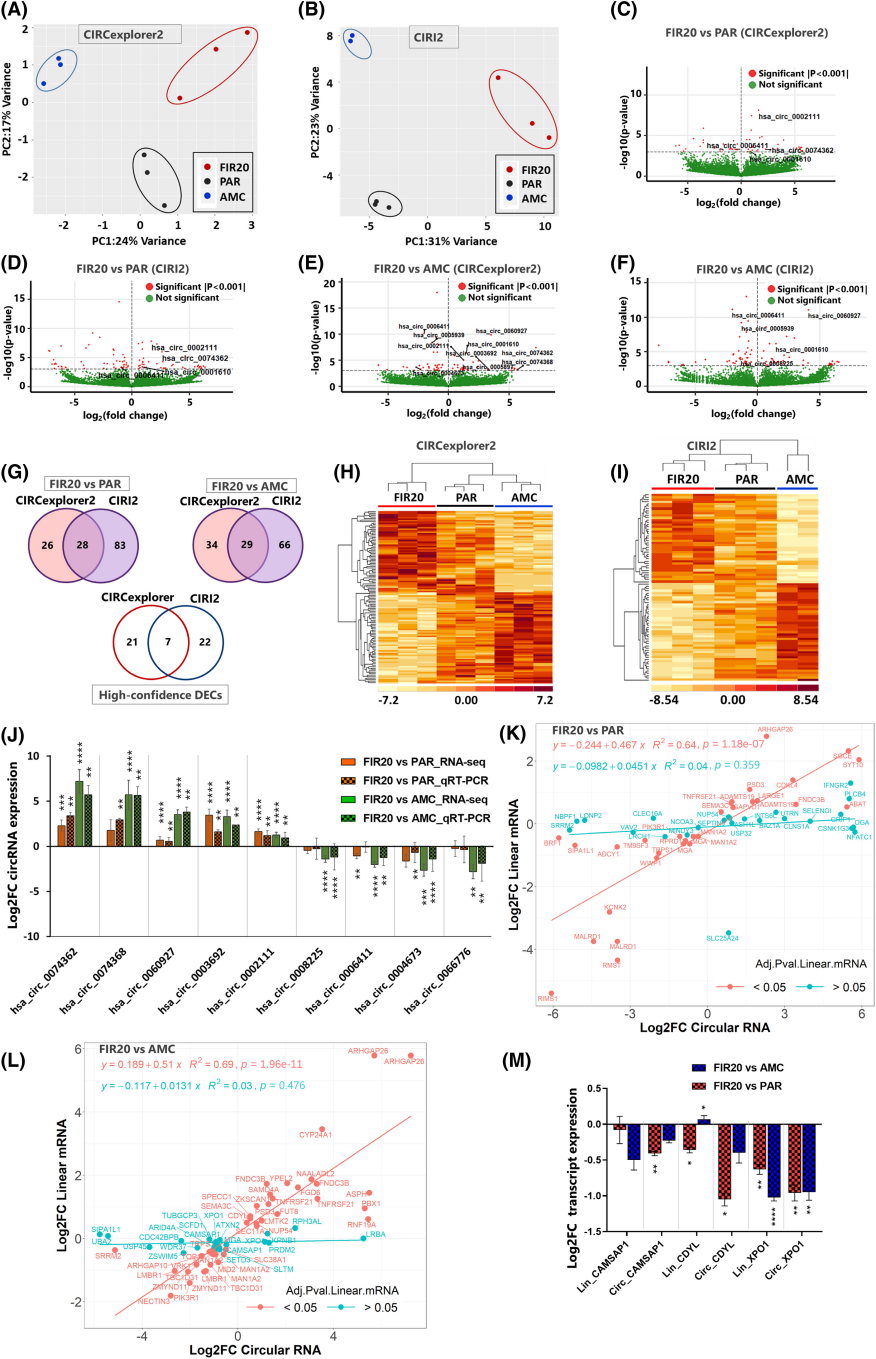
Differential circRNA expression in FIR20 cell lines *vs*. controls. Principal component analysis shows clustering of genes by cell type based on (A) CIRCexplorer2 and (B) CIRI2 detection algorithms. Volcano plots representing DECs detected in FIR20 *vs*. PAR by (C) CIRCexplorer2 and (D) CIRI2, respectively, as well as DECs detected in FIR20 *vs*. AMC by (E) CIRCexplorer2 and (F) CIRI2, respectively. Red dots indicate significant DECs, *P* value ≤ 0.001 and green dots represent nonsignificant DECs. (G) Total DEC transcripts detected by CIRCexplorer2 and CIRI2 algorithms in FIR20 *vs*. PAR (left), FIR20 *vs*. AMC (right) and high‐confidence DECs (bottom) detected by both algorithms (common in both FIR20 *vs*. PAR and FIR20 *vs*. AMC). Heat maps showing hierarchical clustering of samples based on expression levels of DECs detected by (H) CIRCexplorer2 and (I) CIRI2 respectively. Significance given by *P* value ≤ 0.001. CIRI2 failed to detect any circRNA in one sample from the AMC group. (J) Results of qPCR validation of top DECs in the extended signature showing concordance with RNA‐seq results from CIRCexplorer2 plotted side‐by‐side. Data are presented as mean ± (1.96 SE) of three biological replicates. **P < 0.005, ***P = 0.0001, ****P < 0.0001. (K, L) correlation between the change in expression levels of the DECs (with *P* < 0.001) and their cognate linear mRNAs by regression analysis. The circRNAs plotted are based on CIRCexplorer2 results. The square of the correlation coefficient (*R* ^2^) and *P* value (*P*) are shown. Names of several RNA transcripts are labeled. The red regression line depicts the correlation between DECs and cognate mRNAs with *P*adj < 0.05 and the cyan line depicts the relationship between DECs and cognate mRNAs with *P*adj > 0.05. Lower correlation is observed between DECs and cognate linear mRNAs with *P* values > 0.05. (M) Results of qPCR analysis of the differential expression of 3 DECs and their cognate linear mRNAs confirms that not all circRNAs are regulated at the level of their host gene expression **P < 0.001, ****P < 0.0001 by one‐way ANOVA with holm‐Sidak's multiple comparisons test. Data are presented as mean ± (1.96 SE) of three biological replicates. FC, fold change. [Colour figure can be viewed at wileyonlinelibrary.com]

Having established a clear transcriptional shift in FIR20 cells, we next asked whether this was accompanied by changes in circRNA expression. We used two different high performing circRNA prediction algorithms in other to exclude algorithm‐specific false positives [67]. These were CIRCexplorer2 [39] which requires mandatory gene annotation input, and the default *de novo* prediction algorithm CIRI2 [40]. The generated data were then used as input to identify differentially expressed circRNAs (DECs) between cell lines using DeSeq2 [42]. PCA showed good clustering of replicate gene set signatures by cell type (Fig. 6A,B). CIRCexplorer2 identified 28 DECs and CIRI2 29 DECs in the core signature, of which 7 were shared (Fig. 6C–I, Table S6). The 7 common DECs have also previously been reported in relation to cancer development (Table S7) [68] and were therefore considered as *high‐confidence* DECs. We validated the expression of 9 DECs (5 up‐regulated, 4 down‐regulated) which exhibited the highest fold change in the extended signature using qPCR (Fig. 6J, Data S6). Next, we examined whether change in level of expression of the DECs correlate with those of their cognate linear mRNAs using linear regression analysis. A moderate correlation was observed between the expression levels of circRNAs and their cognate mRNAs (linear regression *P* < 0.05 (Fig. S4). The correlation was even lower when only cognate mRNAs with padjusted values > 0.05 were considered (Fig. 6K,L). These results indicate that some circRNAs were regulated independently of the levels of their cognate linear mRNAs and aligns with the notion that circRNAs are not simply splicing byproducts [69]. We experimentally examined the change in expression levels of the circular and linear transcripts of CAMSAP1, CDYL and XPO1 using qPCR. These transcripts were selected based on previous report of their expression in human cancer cells [70]. The results were consistent with our observation that some circRNAs are regulated independent of the levels of their linear mRNAs (Fig. 6M).

### 3.7 DECs compete with miRNAs to regulate radiation‐activated cancer stem cell‐like signaling pathways in FIR20 cells

**Fig. 7:**
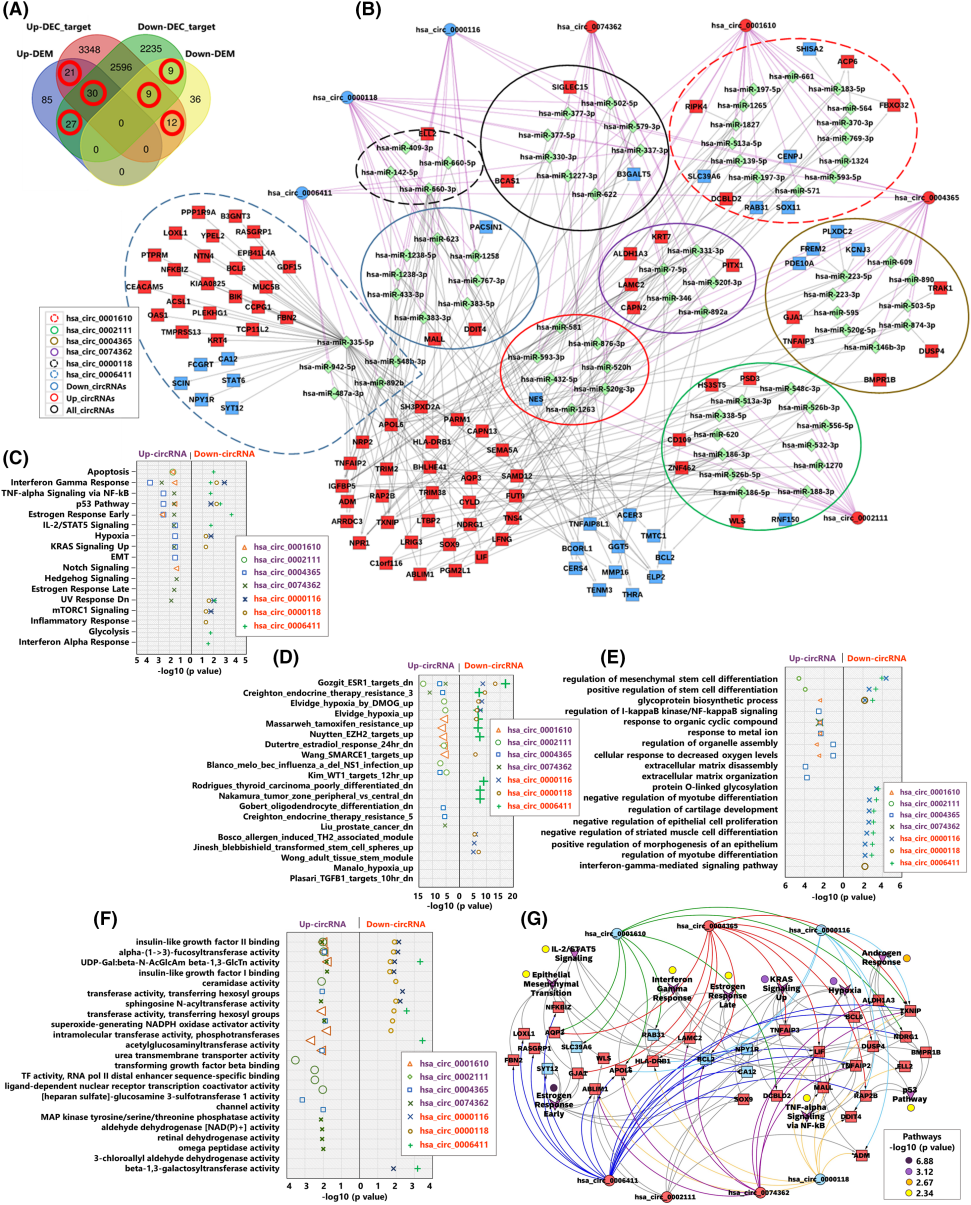
Identification of circRNA‐miRNA‐mRNA regulatory network in FIR20 cells. (A) Venn diagram showing the predicted downstream mRNA targets of the 7 high‐confidence DECs overlapped with the core signature genes. A total of 108 core signature genes (circled red) overlapped with the predicted DEC target mRNAs. (B) High‐confidence ceRNA regulatory network in FIR20 cells based on (A). The network consists of the 7 DECs, 72 predicted miRNAs and their 108 targets from the FIR20 core signature genes. Shapes: Circle = circRNA, diamond = miRNA, and square = mRNA. Shape color: Red = upregulated, blue = downregulated and green = miRNA. Line color: Purple = circRNA‐miRNA interaction, gray = miRNA‐mRNA interaction. Note that miRNAs were identified based on prediction analysis hence their direction of expression is unknown. Ellipses show clusters of miRNAs and mRNAs that are: Unique or shared targets of the individual DECs. Functional enrichment analysis of the independent subsets of up‐ and down‐regulated DEMs predicated as targets of each DEC was performed querying (C) MSigDB hallmark pathways and (D) curated gene sets (E) GO biological process and (F) GO molecular function. (C–F) font color: Purple = upregulated DEC and orange = down‐regulated DEC. (G) circRNA‐mRNA‐pathway network for top 10 enriched Hallmark pathways based on all 108 target core signature genes present in the ceRNA network. TF, transcription factor; UDP‐gal:Beta‐N‐AcGlcAm beta‐1,3‐GlcTn, UDP‐galactose:Beta‐N‐acetylglucosamine beta‐1,3‐galactosyltransferase. [Colour figure can be viewed at wileyonlinelibrary.com]

CircRNAs negatively regulate miRNA activity through their sponge‐effect [18, 19]. We used circinteractome web tool [46] to predict miRNA targets of the 7 high‐confidence DECs. A total of 102 unique target miRNAs were identified, for which 8287 target mRNAs were then predicted using the validated target module of miRWalk [47]. The predicted target mRNAs were intersected with the 229 core signature genes (Fig. 7A) and an overlap of 47.2% (108 DEMs) was obtained. Using this information, we compiled a high‐confidence ceRNA network (Fig. 7B, Data S7A,B), consisting of 7 circRNAs, 72 miRNAs and 108 mRNAs (all core signature genes). The highest number of unique circRNA‐miRNA‐mRNA interactions was related to down‐regulated hsa_circ_0006411 and hsa_circ_0000118, followed by upregulated hsa_circ_0002111 and hsa_circ_0004365. The microRNA, hsa‐miR‐335‐5p, a unique target of the downregulated circPIK3R1_hsa_circ_0006411 had the highest number of unique interactions (18%). The considerable overlap of the core signature genes with predicted targets of dysregulated DECs suggest their involvement in the transcriptional regulation thereof and hence in the radio‐adaptive phenotype of the FIR20 cells. Very much in line with core signature pathway analysis, miRNA targets of the DECs were found to be involved in various cancer‐promoting processes including, but not limited to, regulation of treatment resistance (chemotherapy, endocrine and radiation therapy) and stem cell phenotype (Table S8). This support from the literature was further confirmed by functional enrichment analysis of the subset of 108 core signature genes regulated by DECs (Fig. 7C–F, Fig. S4) and all the mirWalk predicted mRNA targets (Fig. S5).

**Fig. 8:**
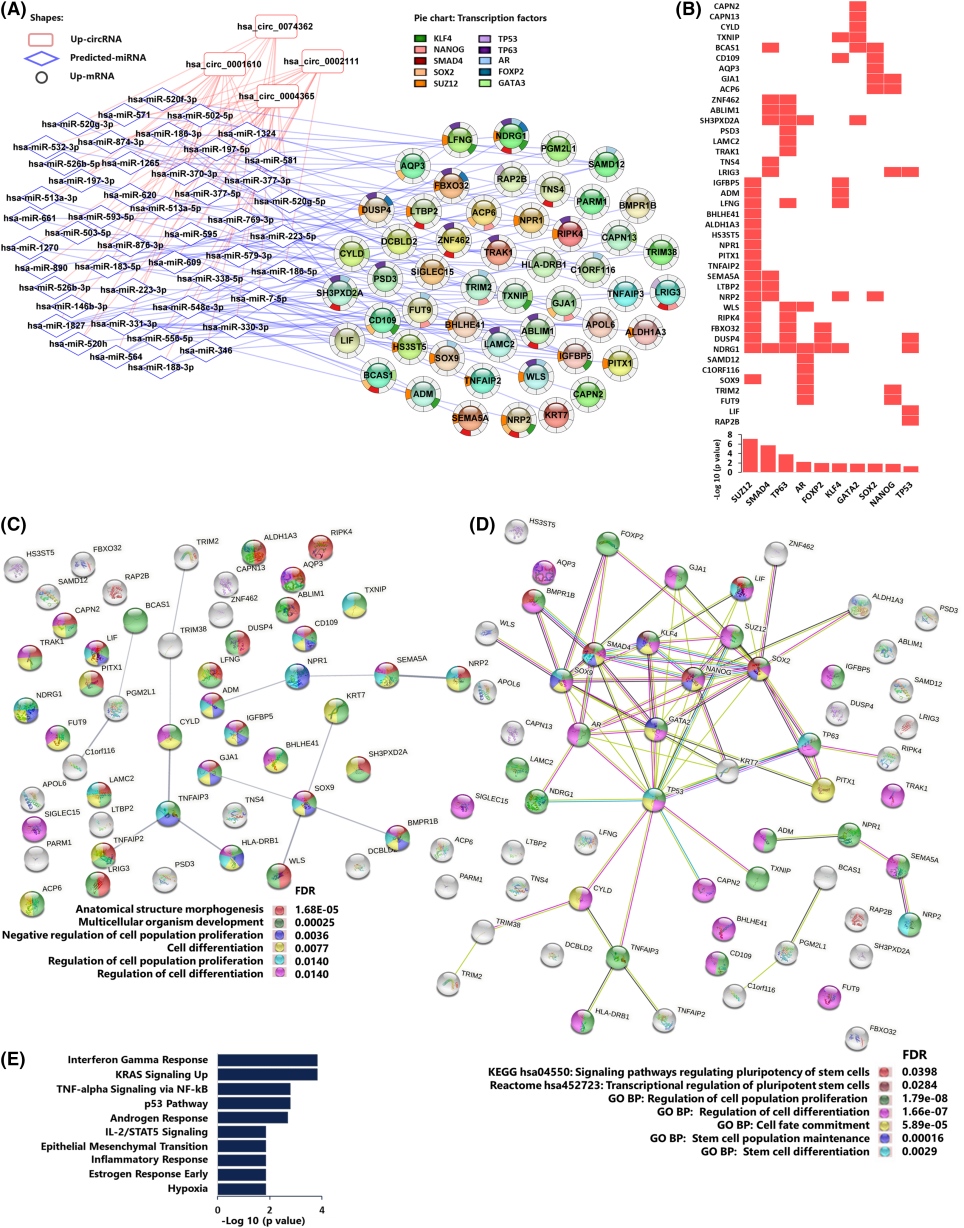
Upregulated DECs are predicted positive regulators of core signature genes involved in stem cell‐like mechanisms in FIR20 cells. (A) ceRNA network of the up regulated DECs and their concordant 51 upregulated target core signature genes. Shapes: DEC = rounded rectangle, miRNA = diamond, target core signature gene = circle. Red lines correspond to circRNA‐miRNA interactions and blue lines depict miRNA‐mRNA interactions. The significant transcription factor regulators of the mRNAs extracted with Enrichr are shown as donut charts around the genes in (A) and also presented in (B) in a heat map (top) and bar chart (bottom). (C) Protein–protein interaction between the protein product of the 51 upregulated core signature genes in the ceRNA network visualized using STRINGDB [45]. Edges indicate both functional and physical PPIs, edge width indicates STRINGDB confidence rank from 0 to 1. Interactions with ‘medium’ confidence score ≥ 0.4 are shown. Pie chart colors correspond to the enriched GO biological processes associated with the network after removing redundancy. (D) the 51 genes were visualized with their regulatory pluripotency‐associated TFs using STRINGDB to identify key associations. Color corresponds to the enriched KEGG and Reactome pathways associated with the PPI network. (E) Enriched MSigDB Hallmark pathways associated with the 51 upregulated target core signature genes in the ceRNA network. [Colour figure can be viewed at wileyonlinelibrary.com]

We performed enrichment analysis of the subset of 51 upregulated core signature genes that were targets of upregulated DECs (DEC‐up_DEM‐target up) and found that many which were regulated by the master pluripotency factors (SOX2 and NANOG) were also retained in this subset and were involved in GO biological processes related to development and cell differentiation (Fig. 8A–D, Data S8). Analysis of potential protein–protein interaction (PPI) between the transcription factors and the upregulated ceRNA‐associated core signature genes also indicated the involvement of LIF and BMPR1B in signaling pathways regulating pluripotency (Fig. 8E). Furthermore, the positively regulated ceRNA‐associated core signature genes were involved in diverse Hallmark pathways (Fig. 8E). These results further highlight the stemness phenotype of the FIR20 cells and the potential role of the DECs as positive regulators of the stem cell‐like mechanisms in the radio‐adapted cells.

## 4 Discussion

Tumour radioresistance remains a clinical challenge that limits the outcome of radiotherapy in breast cancer treatment. To study radioresistance in more detail, we exposed ER‐positive MCF7 breast cancer cells to fractionated 2‐Gy doses of X‐rays to a cumulative of 20 Gy. Based on in‐depth, *in silico* analyses, we established a ceRNA network governing the radiation adaptive response in the fractionally irradiated cells. We find that this network describes a transcriptional rewiring that is characterized by slower growth, increased radioresistance, conversion to a basal‐like subtype, MAPK signaling; and cross‐resistance to tamoxifen. We also found an association between core radio‐adaptive signature genes ‐ ceRNA targets inclusive, with treatment outcome in breast cancer patients treated with endocrine therapy, chemotherapy or radiotherapy, raising the translational value of our findings.

Others have reported that fractionated irradiation enriches or selects for radioresistant clones [30, 71, 72]. Gray et al. [30] also reported that radioresistant MCF7 cells exhibit decreased basal proliferation rate and concurrent lower expression of Ki67 and cell cycle genes than their parent cells. Especially, dormant cells are a lingering risk of tumour recurrence [73, 74]. The quiescent, slow‐growing state has been described as a survival strategy that protects cancer stem cells from anti‐proliferating therapies [75, 76]. There is also evidence for a relative increase in cancer stem cells following RT, chemotherapy and endocrine therapy [11, 12, 13, 77, 78] suggesting that they may be intrinsically resistant and responsible for cancer relapse. Although we observed slower cell proliferation, reduced Ki67 staining and suppression of genes associated with the cell cycle and DNA replication in the FIR20 cells, we did not find the expected change in the proportion of cells distributed across the cell cycle stages. This may be attributed to concurrent changes in nuclear morphology or cell density. Live cell imaging of cell cycle progression may provide more information on differential cycle dynamics in the radioresistant cells and how such changes result in population wide suppression of cell cycle gene expression. The stem cell‐like phenotype of FIR20 cells was accentuated by enrichment in genes associated with blebbishield transformation in cancer stem cells and the expression of stem cell associated genes and proteins, including pluripotency factors.

We observed that the expression of some DECs was independently regulated from their cognate host mRNAs, which suggests that they are independently regulated in the FIR20 cells. According to the circRNA target profiling and pathway enrichment analysis, the high‐confidence DECs could influence the expression of cancer stem cell‐associated core signature genes such as *ALDH1A3, SOX9, CD109, LIF* and *BMPR1* via negative regulation of miRNAs. Among these genes, *ALDH1A3* was identified as a target of hsa_circ_0074362, upstream of hsa‐miR‐7‐5p in our study. Interestingly, Pan and colleagues reported that miR‐7 could inhibit breast cancer growth by suppressing *ALDH1A3* with accompanying decrease in the breast cancer stem cell population [79]. Others have also implicated high ALDH activity involving both ALDH1A3 and ALDH3A1 not only in stemness but also, tumorigenicity and treatment resistance (chemo and radiotherapy) [80, 81, 82, 83]. Other FIR20 enriched transcripts such as *LIF* and *BMPR1* contribute to tumorigenicity and stem cell mechanisms as well [84, 85, 86]. As several of the DEC targets are also targets of key cancer‐promoting transcription factors (*e.g*. OCT4, SOX2, NANOG KLF4 and SUZ12), it is plausible that transcriptional regulation at the level of both transcription factors and the ceRNA network is involved in the stem cell‐like rewiring of FIR20 cells. In this regard, studies have shown that overexpression of these transcription factors can induced normal somatic cells or cancer cells to treatment resistant cancer stem cells [87, 88, 89]. Aberrant regulation of SUZ12 is also linked to tumour initiation and progression by repressing cell fate regulators to promote a stem cell‐like phenotype [87, 88].

Several of the miRNAs in the ceRNA network have also previously been identified as regulators of stem cell‐like properties and cancer treatment resistance (Table S8). For instance, hsa‐miR‐335‐5p was shown to inhibit tumour re‐initiation and metastasis [90], and to synergize with hsa‐miR‐335‐3p to inhibit oestrogen receptor alpha expression and promote tamoxifen resistance in breast cancer cells [91]. It is also associated with chemosensitivity, reduced tumorigenicity and negative regulation of stem cell‐like properties in osteocarcinoma by targeting OCT4 [92].

Another interesting finding was the basal‐like transcriptional reprogramming of the FIR20 cells. The basal breast cancer subtype is characterized by triple negativity (for ESR1, PgR and HER‐2), expression of EGFR and cytokeratin 5/6 [93, 94, 95] and is a more aggressive subtype; associated with high tumour grade, younger age and poor prognosis, including shorter disease‐free and overall survival [96, 97]. Although ESR1 expression was not significantly different in the FIR20 cells, the confirmed increase in EGFR; MAPK pathway; and the basal‐like, oestrogen signaling‐suppressed gene expression profile of FIR20 cells could contribute to their reduced sensitivity to tamoxifen. As the majority of breast cancers (~ 70%) are ER+, anti‐ER therapies are an important treatment option for a large number of breast cancer patients [98, 99]. Furthermore, RT has been shown to be more effective in ER+ breast cancers especially of luminal A subtypes [23, 24, 25, 26, 27]. Moreover, tamoxifen [100, 101] or fulvestrant may be administered to patients concomitantly or in sequence with RT to further inhibit tumour progression [102], so that a substantial number of breast cancer patients receive both radiation and hormonal therapy [102]. This highlights the impact that subtype plasticity and cross‐resistance to both radiation and endocrine therapy can have on treatment and patient outcome.

Our results on EGFR and MAPK signaling align well with transcriptional changes observed in radioresistant derivatives of MCF7 and ZR751 cell lines [30]. Hyperactivation of MAPK in MCF7 cells results in a progressive change to oestrogen receptor alpha negative phenotype with oestrogen‐independent growth [63]. The lack of expression of hormone receptors by basal‐like breast tumours limits the options for targeted treatment of these subtypes [103]. A similar effect of radiation on oestrogen and oestrogen receptor signaling pathways via pathway crosstalk signaling may be responsible for the luminal‐to‐basal‐like transcriptional rewiring we have observed in FIR20 cells. Additionally, oestrogen is also known to regulate Mki67, MYC [104] and E2F [105]; which are key regulators of cell proliferation, cell cycle progression and DNA synthesis [106] that were downregulated in the FIR20 cells. SMAD4, of which targets are enriched in FIR20 cells, is essential to inhibit ESR1 transcription in breast cancer cell lines [107, 108]. Therefore, transcriptional changes in oestrogen signaling dynamics may have had a significant effect on the cellular rewiring in FIR20 cells and could also have a role in their reduced proliferation. Because of the reliance on subtyping for breast cancer treatment [109], better understanding of the underlying drivers of phenotypic and cellular plasticity could be harnessed to improve composition and timing of combination therapies. We show here that circRNAs may contribute to therapy resistance via competitive regulation of miRNAs and mRNAs to promote acquisition of stem cell‐like features in breast cancer cells. The regulatory role of the ceRNA network could be studied further to identify potential targetable modules and prognostic breast cancer markers.

### 4.1 Limitations

We have only investigated one representative breast cancer cell line and it would be valuable to compare with nonhormone responsive types of breast cancer. The cell model was only irradiated to a cumulative of 20 Gy which falls short of the full clinical regiment which can reach 50 Gy for standard adjuvant RT [110]. However, we found various similarities between this cell model and other models treated with higher radiation doses. We also observed only a small increase in the clonogenic survival and radioresistance of the FIR20 model when compared with those demonstrated in other studies [14, 30]. This could be due to differences in the irradiation schedules and the total dose applied. Additionally, FIR20 cells had a recovery period of 9 weeks before being used for the reported investigations. Therefore, this study does not inform about the characteristics of the cells immediately after fractionated irradiation.

RNA‐seq samples were processed by random priming, after ribosomal RNA depletion without enrichment for linear or circRNAs. Nevertheless, similar methods have been used by others and proved to detect linear and circularly ordered exons at comparable levels [111]. To minimize bias in transcript detection, we used separate algorithms (see Section 2) to independently identify linear and circular splicing events. The shared output of two circRNA detection algorithms was also used to increase reliability and exclude false‐positive predictions [67]. As DEC and miRNA targets were based on *in silico* predictions, functional analyses are needed to validate their dysregulation and functional relevance.

## 5 Conclusions

In conclusion, we have established for the first time a comprehensive, high‐confidence ceRNA network governing the adaptive response to fractionated irradiation in MCF7 cells. Our results indicate a transcriptional rewiring towards a stem‐like phenotype that shows features of cross‐resistance to radio‐ and chemotherapy. Some of these changes may be driven by independently regulated circRNAs, which exposes them as possible targets for novel combined anticancer regimen for hormone receptor‐positive breast tumours.

## 7 Conflict of interest

The authors declare no conflict of interest. The funder had no role in designing the study; collecting, analyzing or interpreting the data; or in writing the manuscript and making the decision to publish the results.

## 8 Author contributions

RQ conceived the study. AI, RQ, WHDV and KL designed the experiments. AI, AJ, AC and RQ performed the experiments. AI, BC, JC, RQ, KL and WHDV analyzed the data. RQ and SB acquired funding. AI wrote the draft and revised manuscript. BC, JC, SB, KL, WHDV and RQ revised and corrected the manuscript. All authors read and approved the manuscript.

9

### 9.1 Peer Review

The peer review history for this article is available at https://publons.com/publon/10.1002/1878‐0261.13226.

## Supporting information


**Fig. S1.** Cell cycle analysis.

**Fig. S2.** Gene set enrichment analysis of FIR20 transcriptome.

**Fig. S3.** Tamoxifen cytotoxicity is attenuated in FIR20 cells.

**Fig. S4.** Enrichment analysis of the 108 predicted target genes of the dysregulated DECs.

**Fig. S5.** Functional enrichment analysis of all (8287) miRWalk predicted target genes.

**Table S1.** Primer sequences for qPCR.
**Table S2.** Primary antibodies used for western blotting and immunocytochemistry.
**Table S3.** Growth parameters of FIR20 and control cell lines.
**Table S4.** Radiation sensitivity parameters of cell lines.
**Table S5.** FIR20 enriched genes that overlap with Poste *et al* and Weichselbaum *et al* genes.
**Table S6.** 7 high‐confidence DECs and annotations.
**Table S7.** Published function of DECs.
**Table S8.** Published function and clinical significance of some miRNA targets of the DECs.

**Data S1.** DEMs.xlsx.

**Data S2.** Core signature_Enrichment analysis.xlsx.

**Data S3.** Extended siganture_Transcription factor analysis.xlsx.

**Data S4.** FIR20vsREST transcriptome_GSEA.xlsx.

**Data S5.** Patient data analysis.xlsx.

**Data S6.** DECs.xlsx.

**Data S7A.** ceRNA Network default node.xlsx.

**Data S7B.** ceRNA network.cys.

**Data S8.** CircRNAup_mRNAup_Enrichment_PPI network.xlsx.

## Data Availability

Raw and processed RNA‐seq data are available on GEO, accession, GSE189495.
