# Supplementary figures and images for: Fractionated irradiation of MCF7 breast cancer cells rewires a gene regulatory circuit towards a treatment‐resistant stemness phenotype
Source: Mol Oncol. 2022 Jun 15;16(19):3410–35. doi: 10.1002/1878-0261.13226 (PMC9533694; doi:10.1002/1878-0261.13226)

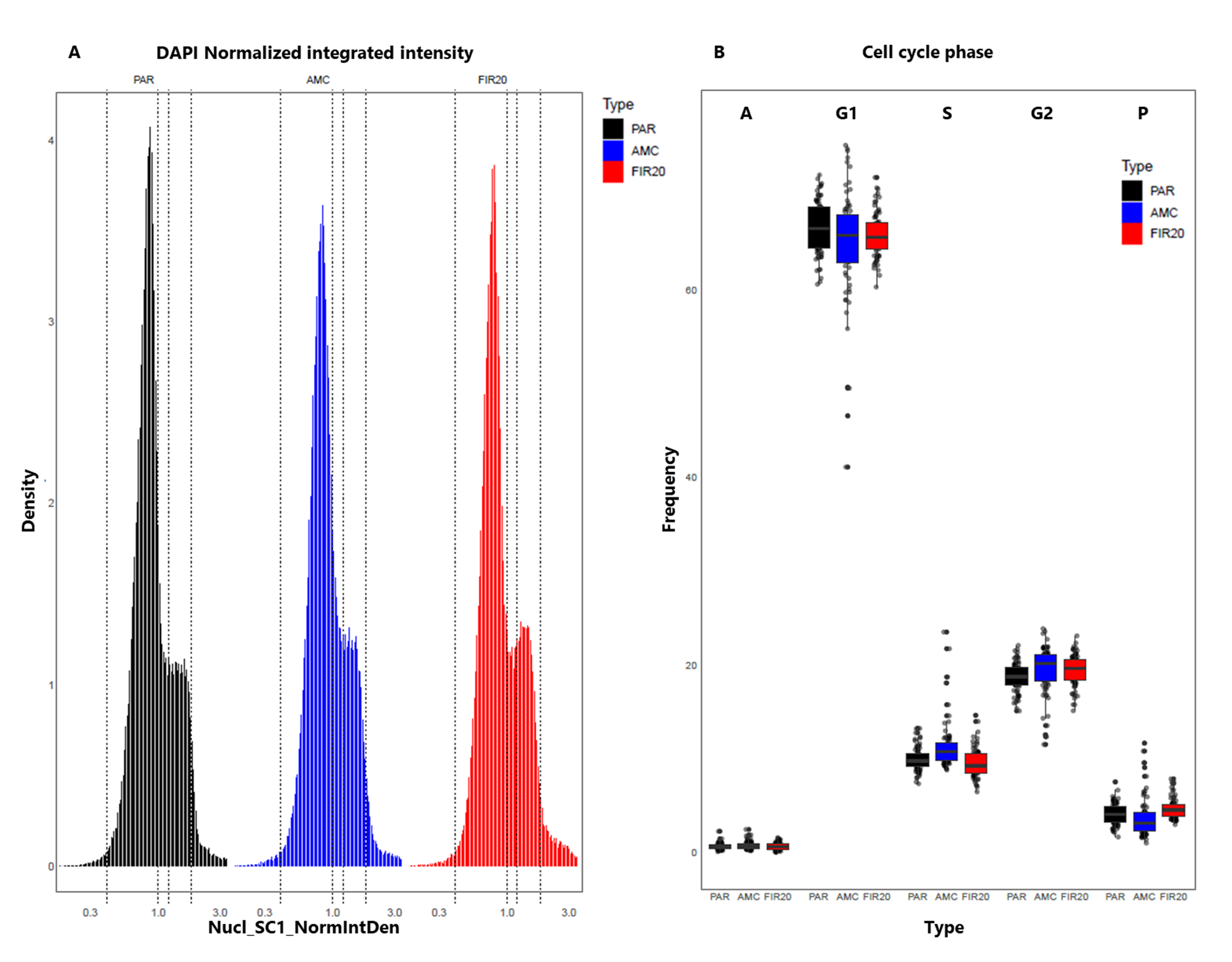

Supplement: Supplementary file 1 — Fig. S1. Cell cycle analysis. [file MOL2-16-3410-s002.tif]

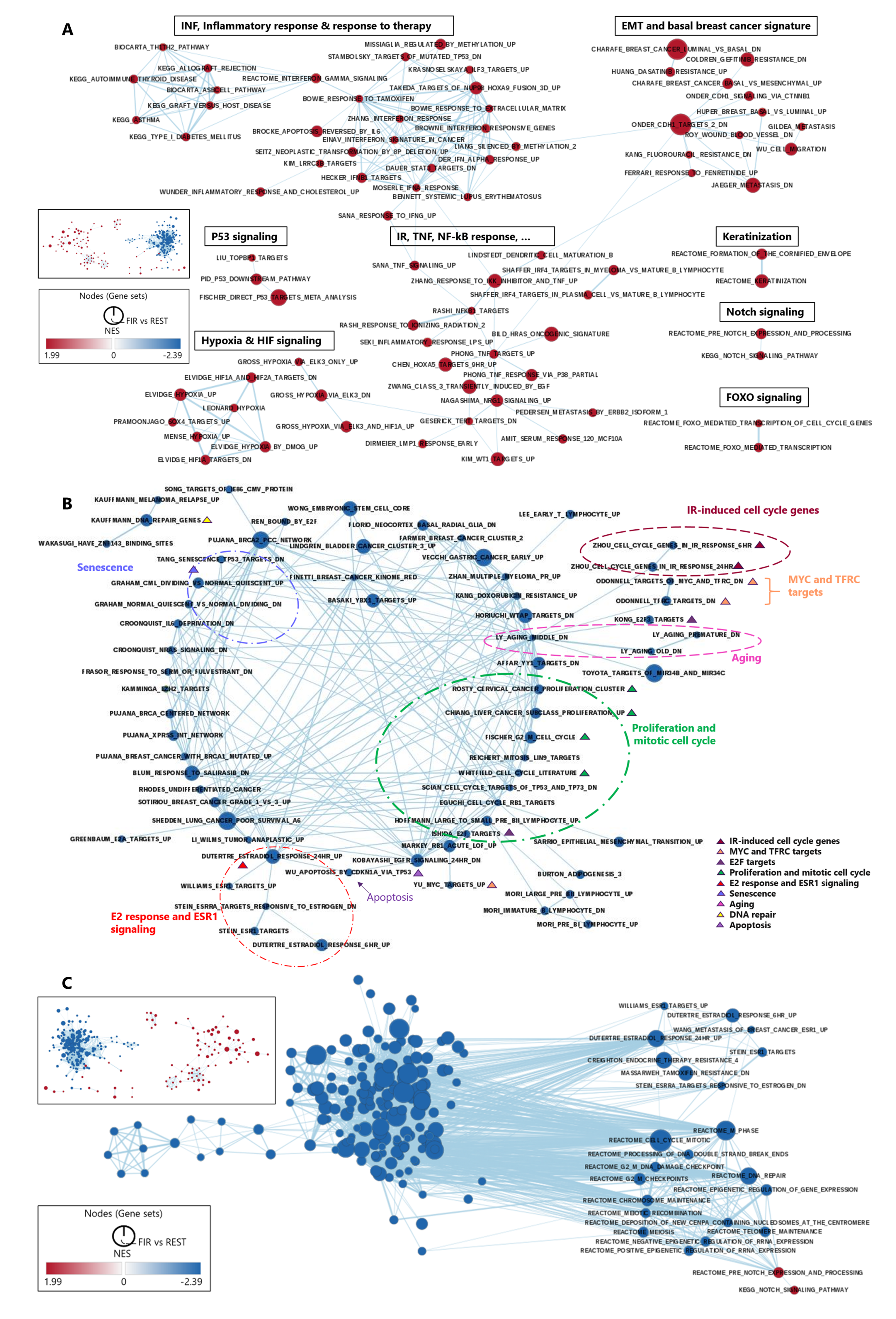

Supplement: Supplementary file 2 — Fig. S2. Gene set enrichment analysis of FIR20 transcriptome. [file MOL2-16-3410-s010.tif]

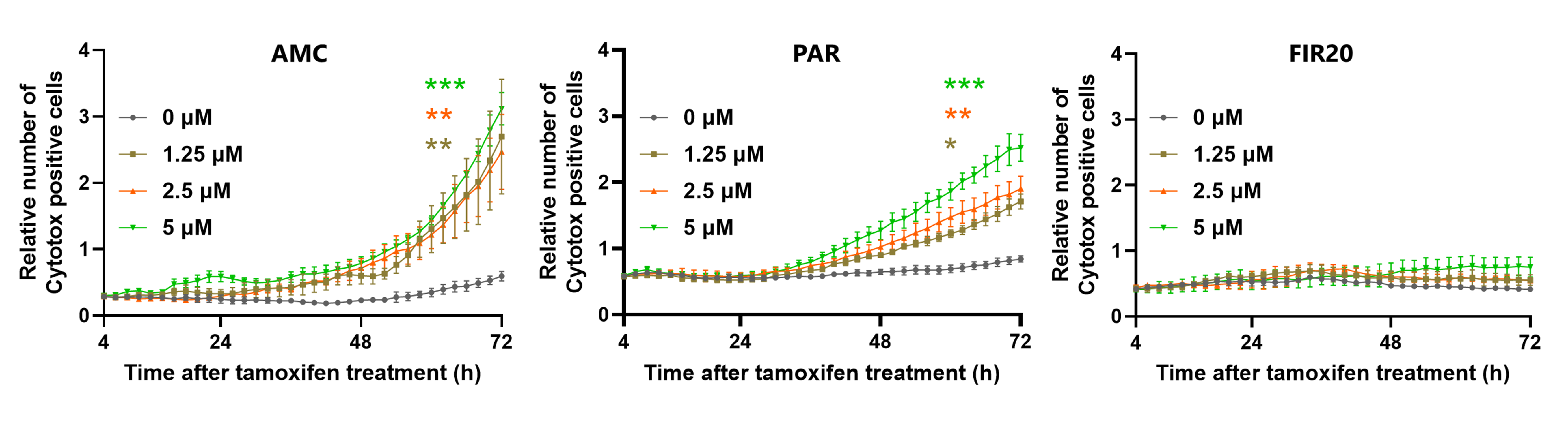

Supplement: Supplementary file 3 — Fig. S3. Tamoxifen cytotoxicity is attenuated in FIR20 cells. [file MOL2-16-3410-s001.tif]

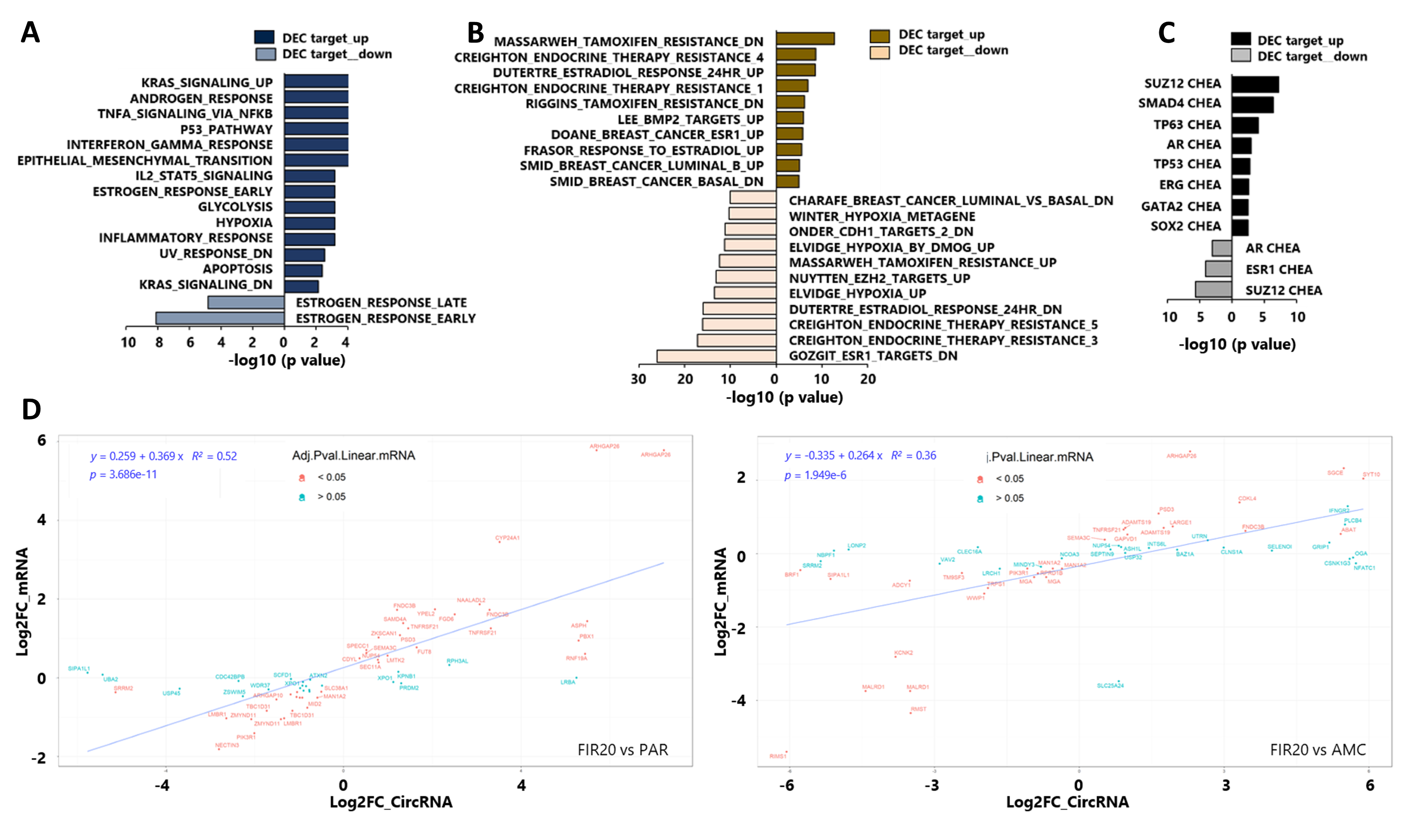

Supplement: Supplementary file 4 — Fig. S4. Enrichment analysis of the 108 predicted target genes of the dysregulated DECs. [file MOL2-16-3410-s015.tif]

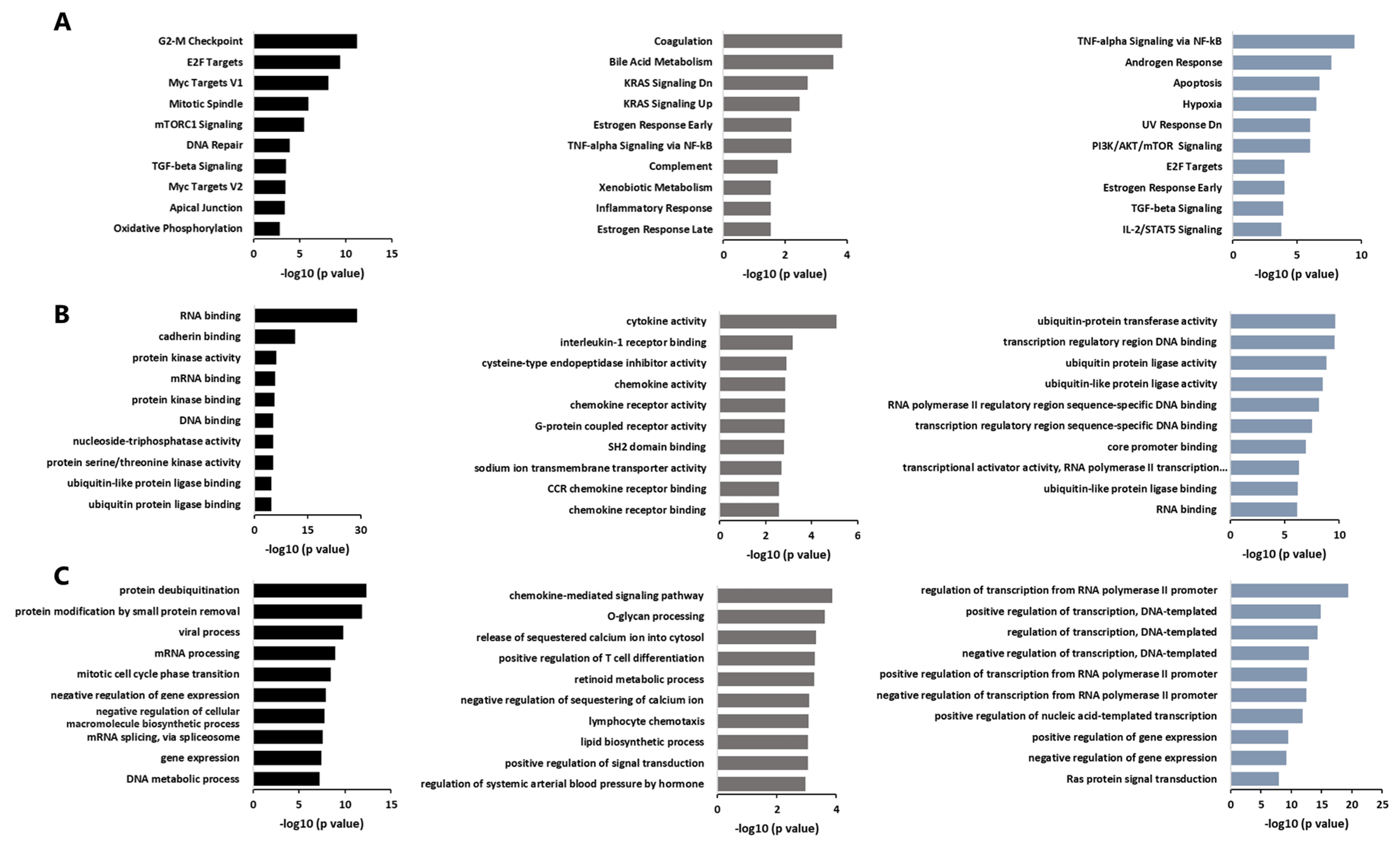

Supplement: Supplementary file 5 — Fig. S5. Functional enrichment analysis of all (8287) miRWalk predicted target genes. [file MOL2-16-3410-s007.tif]
