# Supplementary material for: Fractionated irradiation of MCF7 breast cancer cells rewires a gene regulatory circuit towards a treatment‐resistant stemness phenotype
Source: Mol Oncol. 2022 Jun 15;16(19):3410–35. doi: 10.1002/1878-0261.13226 (PMC9533694; doi:10.1002/1878-0261.13226)
Supplement: Supplementary file 6 — Table S1. Primer sequences for qPCR. Table S2. Primary antibodies used for western blotting and immunocytochemistry. Table S3. Growth parameters of FIR20 and control cell lines. Table S4. Radiation sensitivity parameters of cell lines. Table S5. FIR20 enriched genes that overlap with Poste et al and Weichselbaum et al genes. Table S6. 7 high‐confidence DECs and annotations. Table S7. Published function of DECs. Table S8. Published function and clinical significance of some miRNA targets of the DECs. [file MOL2-16-3410-s014.docx]

# Fractionated irradiation of MCF7 breast cancer cells rewires a gene regulatory circuit towards a treatment-resistant stemness phenotype

**Supplementary tables and figures**

**Fig. S1.** Cell cycle analysis (A) cell cycle histograms generated using DAPI-based cell cycle staging (B) Box plots of cell cycle phases showed no significant difference between the cell lines, n=3.

**Fig. S2.** Gene set enrichment analysis of FIR20 transcriptome. Enrichment analysis of the global transcriptome of *FIR20 vs. REST* was performed using GSEA software and querying the MSigDB C2:Curated gene sets. A network of the overexpressed and under expressed terms are shown as a network in the box (top left) in (A). Red nodes correspond to upregulated gene sets and blue nodes correspond to and downregulated gene sets. For simplification purposes, only the FIR20 enriched terms within the chemical and genetic perturbations subset of the C2:Curated gene sets collection are shown in the main networks corresponding to (A) upregulated gene sets and (B) downregulated gene sets. A threshold of FDR ≤ 0.20 and overlap co-efficient of 0.55 was applied. Singleton gene sets/nodes are not shown. (C) High connectivity between downregulated targets of Notch, estrogen receptor signalling and/or estrogen signalling which are also involved in cell cycle regulation. NES, normalized enrichment score. Networks were created using Cytoscape software [1].

**Fig. S3.** Tamoxifen cytotoxicity is attenuated in FIR20 cells. After 72 h of treatment with 1.25, 2.5 and 5 µM of tamoxifen, there was statistically significant increase in cytotoxicity in PAR and AMC cells compared to untreated (0 µM) controls but not in FIR20 cells. Linear mixed effect model with Tukey’s Honest significant difference test, p≤0.05, ** p ≤ 0.01,*** p ≤0.001, n=3.

**Fig. S4.** Enrichment analysis of the 108 predicted target genes of the 7 dysregulated DECs in FIR20 cells based on (A) MSigDB Hallmarks, (B) MSigDB Curated gene sets, and (C) transcription factor enrichment. Enrichment analysis was performed with Enrichr [2]. Transcription factors were extracted from the *ENCODE and ChIP Enrichment Analysis (ChEA) Consensus TFs from ChIP-X* database [67]. (D) Correlation between the expression levels of DECs and their cognate linear mRNAs from our RNA-seq data depicted by linear regression analysis, FIR20 vs. PAR (left) and FIR20 vs. AMC (right) based on CIRCexplorer2 results. Only DECs with p<0.001 were plotted. Several RNA transcripts are labelled. The square of the correlation coefficient (R2) and p values (p) are shown.

**Fig. S5.** Functional enrichment analysis of all (8287) miRWalk predicted target genes of the dysregulated DECs in FIR20 cells for (A) MSigDB Hallmarks (B) GO molecular function (C) GO biological process. The graphs indicate the significantly enriched terms for Left: upregulated DEC targets, Middle= downregulated DEC targets, Right: All DEC targets.

**Table S1.** Table 1 Primer sequences for qPCR

| Primer | Sequence (5'->3') |
| --- | --- |
| CEACAM5-FW | CAATGGGACTTTCCAGCAATC |
| CEACAM5-RV | GGTGGCTCTGCATAGACTGT |
| CEACAM6-FW | AGTCACCCTGAATGTCCTCTA |
| CEACAM6-RV | CTGGACGGTAATTGGCCTTT |
| COL17A1-FW | CGGACTATTGGGAGCTGACT |
| COL17A1-RV | GACAGTGTAGGCCATCCCTT |
| SOX21-FW | CGAGTGGAAACTGCTCACAG |
| SOX21-RV | CGGGAAGGCGAACTTGTC |
| FBN2-FW | TGAATGTTCCAACACCGTGG |
| FBN2-RV | CACATGCCTGTTCTCTGATCG |
| SOX11-FW | AGGGCGAATTCATGGCTTG |
| SOX11-RV | CGGAGACTGCTCCATGATCT |
| NPY1R-FW | GTTGCCATCATGTGTCTCCC |
| NPY1R-RV | CACATCGCCTCACCAAAGAC |
| ALDH3A1-FW | CTCGGAGCTGAGTGAGAACA |
| ALDH3A1-RV | GCCCGTGTACAGGATATGGT |
| ALDH1A3-FW | GGGAAGCCATTTCTTCATGCT |
| ALDH1A3-RV | GAAGCACACGACGTTGTCAT |
| CD44-FW | ACTGGAACCCAGAAGCACAC |
| CD44-RV | GTTGCCAAACCACTGTTCCT |
| CD109-FW | CTGGGTACGTCCGGTTACAC |
| CD109-FW | TGTCCTGGAAGCAGAAGGAG |
| SOX9-FW | GAAGGACCACCCGGATTACA |
| SOX9-RV | CCTTGAAGATGGCGTTGGG |
| hsa_circ_ 0074362-FW | AGCTTTGTCGGAAGAGGACC |
| hsa_circ_ 0074362-RV | TCAAGTGTTTTTCTAAGATGCCACA |
| hsa_circ_ 0074368-FW | CTCGGGTCTCTGAAATCCAC |
| hsa_circ_ 0074368-RV | TTATGCTGAAGCCAATGCTG |
| hsa_circ_ 0060927-FW | AGCCTGTTGAGATGCTACACT |
| hsa_circ_ 0060927-RV | GTTGTCCAGCTTCATCACTTCC |
| hsa_circ_ 0003692-FW | TAAGCTGGCACATAGGAGCA |
| hsa_circ_ 0003692-RV | TCGGGTGATGTAGGTTGACA |
| hsa_circ_ 0002111-FW | ACCTGTCAGGAGTTCATTGC |
| hsa_circ_ 0002111-RV | ATGAGCTTCTAGCCGTGTTG |
| hsa_circ_ 0008225-FW | TTGCCTGGAGAGGTGTTGAT |
| hsa_circ_ 0008225-RV | ATCTCAATGGCTGCCCAAAGA |
| hsa_circ_ 0006411-FW | CTCGGCCTCTTCCTGTTG |
| hsa_circ_ 0006411-RV | GCCCAATCCTTTCTGACACT |
| hsa_circ_ 0004673-FW | ACTCCCAAGAAAGATGACACAACT |
| hsa_circ_ 0004673-RV | TGACACTGCGAGAGTGAACG |
| hsa_circ_ 0066776-FW | TTGTCCATCCATTGACTTTCAATTA |
| hsa_circ_ 0066776-RV | TACTGCTGTGACATGTGGCT |
| *CAMSAP1 circular-FW | AGTGCCTCGAAAGAACTTC |
| *CAMSAP1 circular-RW | TCCTGCTCATACTGGTCAA |
| *CDYL circular-FW | CTTAGCTGTTAACGGGAAA |
| *CDYL circular-RV | CTGTTGAAGTCGTGGATGT |
| *XPO1 circular-FW | CCAAGGAACCAGTGCGAAG |
| *XPO1 circular-RV | GAAATCAAGCAGCTGACGA |
| *CAMSAP1 linear-FW | AAGGCTGTCACAGGCACTAC |
| *CAMSAP1 linear-RV | CGCTGATCAGGGATGTCCTC |
| *CDYL linear-FW | CTCGTGCAATCCAGTTGTGC |
| *CDYL linear-RV | ACCTCACACTCCCTCTCGTT |
| *XPO1 linear-FW | TGATCCACAGATGGTCGCTG |
| *XPO1 linear-RV | GGTTCTCTAGCAGCTGGGAC |

*Linear and cognate circRNA primers taken from [3].

**Table S2:** Primary antibodies used for western blotting and immunocytochemistry

| **Antigen** | **Species/clonality** | **Company** | **Catalog number** | **Concentration** |
| --- | --- | --- | --- | --- |
| Ki67 | Rabbit Polyclonal | Abcam | ab15580 | 1/200 |
| EGFR | Rabbit monoclonal | Cell Signaling | 4267 | 1/1000 (WB) |
| ERK1/2 | Rabbit monoclonal | Cell Signaling | 4695 | 1/2000 (WB) |
| Phospo ERK1/2 | Rabbit monoclonal | Cell Signaling | 4370 | 1/2000 (WB) |
| ESR1 | Rabbit polyclonal | Merck Millipore | 06-935 | 1/2000 (WB) 1/500 (ICC) |
| OCT4. | Rabbit polyclonal | Abcam | ab19857 | 1/2000 (WB) |
| CD44 | Mouse monoclonal | Cell Signaling | 3570 | 1/2000 (WB) |
| SOX2 | Rabbit polyclonal | Abcam | ab97959 | 1/2000 (WB) |
| SOX9 | Rabbit Polyclonal | Abcam | ab5535 | 1/1000 (ICC) |

**Table S3:** Growth parameters of FIR20 and control cell lines.

| Cell line | Doubling time (Dt) | 95% Confidence interval | R squared |
| --- | --- | --- | --- |
| FIR 20 | 34.74 | 34.11 to 35.40 | 0.9402 |
| AMC | 27.21 | 27.04 to 27.39 | 0.994 |
| PAR | 28.01 | 27.51 to 28.54 | 0.9612 |

**Table S4:** Radiation sensitivity parameters of cell lines.

| Cell line | D_10_ | $\hat{\varphi}_{2}$ | SE $\hat{\varphi}_{2}$ | SF_2_ ± SE | α | β | α/β |
| --- | --- | --- | --- | --- | --- | --- | --- |
| FIR 20 | 3.70 | 1.843 | 0.036 | 0.43 ± 0.03 | 0.14 | 0.13 | 1.09 |
| AMC | 3.37 | 1.642 | 0.052 | 0.34 ± 0.004 | 0.32 | 0.11 | 2.98 |
| PAR | 3.33 | 1.583 | 0.058 | 0.32 ± 0.03 | 0.48 | 0.07 | 6.86 |

**Table S5:** FIR20 enriched genes that overlap with Post et al and Weichselbaum genes

Lists of the 21 genes increased in the extended signature out of the 94 genes identified by Post et al with increased expression levels in both MCF-7^TAM^ and MCF-7^RT^ compared to MCF-7^WT^ [4]. Below this are the 11 genes of Weichselbaum et al. [5] that were also differentially induced in the extended signature. For each gene, the log2 fold increase in expression levels in FIR20 relative to PAR or AMC is shown. Genes that are present in both published data sets are written in bold font.

|  | Gene Name | Log2 Fold increase FIR vs PAR | pvalue vs PAR | padj vs PAR | Log2 Fold increase FIR vs AMC | pvalue vs AMC | padj vs AMC | Correlated in patients | INF-related |
| --- | --- | --- | --- | --- | --- | --- | --- | --- | --- |
|  | Post et al genes induced in tamoxifen resistant and radioresistant cells | | | | | | | | |
| 1 | IGFBP3 | 2.22 | 1.41E-04 | 2.82E-03 | 5.06 | 7.21E-11 | 5.51E-09 | No | No |
| 2 | CRYBG3 | 2.23 | 2.28E-04 | 4.19E-03 | 2.92 | 6.71E-06 | 1.60E-04 | No | No |
| 3 | CEMIP | 3.16 | 4.39E-06 | 1.62E-04 | 3.92 | 2.15E-08 | 9.51E-07 | No | No |
| **4** | **OAS1** | 3.42 | 4.81E-07 | 2.43E-05 | 4.14 | 3.46E-08 | 1.46E-06 | Yes | Yes |
| 5 | PARP9 | 1.14 | 2.54E-03 | 2.81E-02 | 1.83 | 2.89E-06 | 7.70E-05 | No | No |
| 6 | IFIH1 | 2.59 | 4.31E-05 | 1.05E-03 | 2.74 | 2.26E-05 | 4.44E-04 | No | No |
| 7 | APOL6 | 2.23 | 2.96E-03 | 3.15E-02 | 4.79 | 8.04E-06 | 1.85E-04 | Yes | Yes |
| 8 | PARP14 | 1.53 | 1.81E-05 | 5.24E-04 | 1.71 | 2.18E-06 | 6.01E-05 | No | No |
| 7 | IL1R1 | 1.66 | 1.41E-04 | 2.81E-03 |  |  |  | No | No |
| 10 | IL6ST | 1.10 | 2.17E-06 | 9.00E-05 |  |  |  | No | No |
| **11** | **MX1** | 2.76 | 1.17E-03 | 1.54E-02 |  |  |  | Yes | Yes |
| 12 | TSPAN12 | 6.00 | 5.93E-05 | 1.37E-03 |  |  |  | No | No |
| 13 | **BST2** |  |  |  | 2.94 | 5.90E-03 | 4.22E-02 | Yes | Yes |
| 14 | DDX60 |  |  |  | 3.38 | 3.60E-03 | 2.88E-02 | Yes | Yes |
| 15 | DHRS2 |  |  |  | 1.44 | 2.83E-04 | 3.67E-03 | No | No |
| **16** | **IFIT3** |  |  |  | 5.68 | 6.59E-04 | 7.19E-03 | Yes | Yes |
| 17 | ISG15 |  |  |  | 1.00 | 5.87E-04 | 6.61E-03 | No | No |
| 18 | LIMCH1 |  |  |  | 2.30 | 4.05E-05 | 7.27E-04 | No | No |
| 19 | MDK |  |  |  | 1.91 | 1.34E-05 | 2.87E-04 | No | No |
| 20 | NAV3 |  |  |  | 5.06 | 6.20E-04 | 6.89E-03 | No | No |
| 21 | PARP10 |  |  |  | 1.96 | 2.78E-05 | 5.31E-04 | No | No |
|  | Weichselbaum et al. IRDS genes | | | | | | | | |
|  | **OAS1** | 3.42 | 4.81E-07 | 2.43E-05 | 4.137342721 | 3.46E-08 | 1.46E-06 |  |  |
|  | ALDH3A1 | 4.16 | 4.28E-04 | 7.09E-03 | 2.680360837 | 6.75E-03 | 4.69E-02 |  |  |
|  | **MX1** | 2.76 | 1.17E-03 | 1.54E-02 |  |  |  |  |  |
|  | ROBO1 | 1.28 | 4.43E-18 | 1.71E-15 |  |  |  |  |  |
|  | OASL | 2.46 | 1.01E-03 | 1.37E-02 |  |  |  |  |  |
|  | **BST2** |  |  |  | 2.938997975 | 5.90E-03 | 4.22E-02 |  |  |
|  | **IFIT3** |  |  |  | 5.680406951 | 6.59E-04 | 7.19E-03 |  |  |
|  | CD59 |  |  |  | 1.383952918 | 2.37E-15 | 3.45E-13 |  |  |
|  | PLSCR1 |  |  |  | 1.461603208 | 6.37E-03 | 4.49E-02 |  |  |
|  | TIMP3 |  |  |  | 1.258802762 | 2.59E-05 | 4.99E-04 |  |  |
|  | TRIM14 |  |  |  | 1.669694059 | 1.77E-13 | 2.00E-11 |  |  |

**Table S6:** 7 high-confidence DECs and annotations.

| CircRNA ID | gene symbol | Position | Strand | CircRNA study | Expression in FIR20 |
| --- | --- | --- | --- | --- | --- |
| hsa_circ_0002111 | PSD3 | chr8:18622958-18662408 | - | [6–9] | Up |
| hsa_circ_0074362 | ARHGAP26 | chr5:142264862-142311690 | + | [7–9] | Up |
| hsa_circ_0001610 | TNFRSF21 | chr6:47251673-47254331 | - | [6–8, 10] | Up |
| hsa_circ_0004365 | SEMA3C | chr7:80418621-80440017 | - | [6–9] | Up |
| hsa_circ_0006411 | PIK3R1 | chr5:67522117-67522837 | + | [6–8] | Down |
| hsa_circ_0000118 | MAN1A2 | chr1:117944807-117963271 | + | [7, 8, 10] | Down |
| hsa_circ_0000116 | MAN1A2 | chr1:117944807-117948267 | + | [7–10] | Down |

**Table S7:** Published function of DECs.

| CircRNA ID | gene symbol | Strand | Expression in FIR20 cells | Cancer | Expression in cancer | Significance | Ref |
| --- | --- | --- | --- | --- | --- | --- | --- |
| hsa_circ_0002111 | PSD3 | - | Up | Papillary Thyroid Cancer (PTC) | Up | Advanced TNM stage, lymph-node metastasis. Promotes PTC. Proliferation, invasion. | [11] |
|  |  |  |  | PTC | UP |  | [12] |
|  |  |  |  | PTC | Up | Associated with the BRAF^V600E^ mutation in PTC | [13] |
|  |  |  |  | PTC | UP |  | [14] |
| hsa_circ_0074362 | ARHGAP26 | + | Up | Gastric cancer | down | Lymphatic metastasis. | [15] |
| hsa_circ_0001610 | TNFRSF21 | - | Up | Endometrial cancer | Up | Promotes EC cell growth, proliferation, colony formation, cell cycle progression, apoptosis, sponges miR-1227 | [16] |
| hsa_circ_0004365 | SEMA3C | - | Up |  | up | Metastasis | [17] |
| hsa_circ_0006411 | PIK3R1 | + | Down | Glioma | down |  | [18] |
| hsa_circ_0000118 | MAN1A2 | + | Down | Cervical Adenocarcinoma | up |  | [19] |
| hsa_circ_0000116 | MAN1A2 | + | Down | Colon cancer |  |  | [20] |

**Table S8:** Published function and clinical significance of some miRNA targets of the DECs.

| miRNA | Predicted circRNA sponge in FIR20:  CircRNA (expression) | Cancer | Expression in cancer | Biological functions/clinical significance in cancer | Ref |
| --- | --- | --- | --- | --- | --- |
| hsa-miR-335-5p | hsa_circ_0006411 (down) | Breast | Down | Inhibits tumor re-initiation and metastasis. | [21] |
| hsa-miR-335-5p | hsa_circ_0006411 (down) | Osteosarcoma | Down | Negatively regulates stem cell-like properties by targeting POU5F1. Increases chemosensitivity, inhibits in vivo tumor formation . | [22] |
| hsa-miR-335-5p | hsa_circ_0006411 (down) | Melanoma |  | Acts as tumor suppressor, attenuates proliferation, migration, and radioresistance by targeting ROCK1. | [23] |
| hsa-miR-335-5p | hsa_circ_0006411 (down) | Small cell lung cancer | Down | Regulates chemo-radioresistance by targeting PARP-1, inhibits cell migration in vitro and tumor growth in vivo. Inhibits cell proliferation, clonality and apoptosis. | [24] |
| hsa-miR-335-5p | hsa_circ_0006411 (down) | Colorectal cancer | Down | Inhibits cell proliferation, migration and invasion through downregulating LDHB | [25] |
| hsa-miR-335-5p | hsa_circ_0006411 (down) | Gastric cancer | Down | Downregulated by long noncoding RNA ZEB1-AS, inhibits tumor proliferation and invasion. | [26] |
| hsa-miR-335-5p | hsa_circ_0006411 (down) | Breast cancer | Down | Suppresses proliferation, migration, and invasion by targeting EphA4. | [27] |
| hsa-miR-335-5p | hsa_circ_0006411 (down) | Breast cancer | Down | Inhibits migration by targeting oncoprotein c-Met. | [28] |
| hsa-miR-335-5p | hsa_circ_0006411 (down) | Triple negative Breast cancer | Down | Increases sensitivity of tumor cells to paclitaxel, cisplatin and doxorubicin, improves the effect of chemotherapy. | [29] |
| hsa-miR-335-5p | hsa_circ_0006411 (down) | Breast cancer | Down | Decreases cell viability, increases apoptosis, upregulates BRCA1. | [30] |
| hsa-miR-335-5p | hsa_circ_0006411 (down) | Breast cancer | Down | Suppresses CDH11, β-catenin and vimentin expression, suppresses CSC-like and metastatic phenotype. | [31] |
| hsa-miR-335-5p | hsa_circ_0006411 (down) | Breast cancer | Down | Sponged by Circ_0007255 to regulate SIX2 expression in breast cancer progression, abrogates the effects of Circ-0007255 on oxygen consumption, colony formation, cell migration and invasion. | [32] |
| hsa-miR-335-5p | hsa_circ_0006411 (down) | Gastric cancer | Down | Inhibits proliferation and migration, suppresses gastric cancer progression by targeting MAPK10. | [33] |
| hsa-miR-335-5p | hsa_circ_0006411 (down) | Colorectal cancer | Upregulated in exosomes | Promotes cell invasion and metastasis by facilitating EMT via targeting RASA1. | [34] |
| hsa-miR-335-5p | hsa_circ_0006411 (down) | Breast cancer | Upregulated in taxanes-resistant breast cancer samples | Increases cell proliferation, reduces apoptosis and sensitivity to paclitaxel and docetaxel, correlates with taxanes-resistance and poor prognosis in breast cancer. | [35] |
| hsa-miR-335-5p | hsa_circ_0006411 (down) | Epithelial ovarian cancer (EOC) | Downregulated in malignant samples | Associated with prognosis, promising predictor of EOC recurrence. | [36] |
| hsa-miR-335-5p | hsa_circ_0006411 (down) | Breast cancer | Higher in normal vs tumor | Synergizes with 335-3p to inhibit estrogen receptor alpha expression and promote tamoxifen resistance. | [37] |
| hsa-mir-660-5p | hsa_circ_0000118  (down) | Breast cancer | up | Associated with tumor development and metastasis, regulates proliferation, migration, and invasion, targets TFCP2. | [38] |
| hsa-mir-502-5p | hsa_circ_0000118 (down)  hsa_circ_0000116 (down)  hsa_circ_0074362 (up) | Breast cancer | down | Enhances apoptosis, inhibits proliferation. regulates TRAF2 | [39] |
| hsa-mir-139-5p | hsa_circ_0001610 (up) | Breast cancer | down | Mediates chemosensitivity to docetaxel, inhibits cell viability, induces apoptosis, causes cell cycle arrest in S phase, inhibits migration and invasion, regulates the expression of Notch1. | [40] |
| hsa-mir-139-5p | hsa_circ_0001610 (up) | Colorectal cancer | down | Reverses CD44+/CD133+-associated multidrug resistance by downregulating NOTCH1. | [41] |
| hsa-mir-139-5p | hsa_circ_0001610 (up) | Nasopharyngeal carcinoma | down | Affects cisplatin sensitivity, counteracts metastasis and chemotherapy resistance, inhibits proliferation, invasion, migration and EMT. | [42] |
| hsa-mir-330-3p | hsa_circ_0000118 (down)  hsa_circ_0074362 (up) | Breast cancer | up | Associated with lower distant relapse-free survival, increases cell invasiveness, metastasis, and aggressiveness. | [43] |
| hsa-mir-330-3p | hsa_circ_0000118 (down)  hsa_circ_0074362 (up) | Breast cancer | up | Associated with prognosis. | [44] |
| hsa-mir-346 | hsa_circ_0074362 (up) | Breast cancer | up | Mediate chemo-sensitivity to docetaxel through targeting SRCIN1. Promotes cell proliferation, colony formation, migration and invasion, reduces apoptosis. | [45] |
| hsa-mir-346 | hsa_circ_0074362 (up) | Hepatocellular carcinoma (HCC) | up | Promotes proliferation and inhibits apoptosis. Promotes HCC progression by Suppressing BRMS1 expression. | [46] |
| hsa-mir-223-3p | hsa_circ_0074362 (up) | Head and neck squamous cell carcinoma | up | Inhibits angiogenesis and promotes resistance to cetuximab | [47] |
| hsa-mir-433-3p | hsa_circ_0000118 (down)  hsa_circ_0000116 (down) | Glioma | down | Suppresses cell growth and enhances chemosensitivity by targeting CREB. | [48] |
| hsa-mir-338-5p | hsa_circ_0002111 (up) | Esophageal squamous cell carcinoma | down | Inhibits cell proliferation, colony formation, migration and cisplatin resistance by targeting FERMT2. | [49] |
| hsa-mir-520g | hsa_circ_0004365 (up)  hsa_circ_0074362 (up) | Breast cancer | up | Associated with mammary gland invasion, lymph node metastasis and low differentiation . | [50] |
| hsa-mir-876-3p | hsa_circ_0004365 (up)  hsa_circ_0001610 (up) | Gastric cancer | down | Inhibits cisplatin resistance and cell stem cell-like properties by targeting TMED3. | [51] |
| hsa-mir-1258 | hsa_circ_0000118 (down)  hsa_circ_0000116 (down) | Breast cancer | down | Inhibits heparanase in vitro cell invasion and experimental brain metastasis. | [52] |
| hsa-mir-183-5p | hsa_circ_0001610 (up) | Breast cancer | up | Promotes cell proliferation, inhibits apoptosis by targeting the PDCD4. | [53] |
| hsa-mir-383 |  | Breast cancer | down | Regulates apoptosis through targeting Gadd45g. | [54] |
| hsa-mir-383-5p | hsa_circ_0000118 (down)  hsa_circ_0000116 (down) | Ovarian cancer | down | Suppresses proliferation, enhances chemosensitivity by targeting TRIM27. | [55] |
| hsa-mir-526b | hsa_circ_0002111 (up) | Breast cancer | up | Increases cellular migration, invasion, EMT phenotype, enhances tumorsphere formation in vitro, is regulated by COX-2 by EP4 activation. | [56] |
| hsa-mir-526b | hsa_circ_0002111 (up) | Breast cancer | up | Induces oxidative Stress. | [57] |
| hsa-mir-513a-5p | hsa_circ_0001610 (up) | Osteosarcoma | down | Induces/regulates radiosensitivity by targeting APE1. | [58] |
| hsa-mir-186 | hsa_circ_0002111 (up) | Breast cancer | down | Inhibits proliferation, migration, and EMT by targeting Twist1. | [59] |
| hsa-mir-383-5p | hsa_circ_0000118 (down) | Ovarian cancer | down | Suppresses proliferation and enhances chemosensitivity by targeting TRIM27. | [55] |
| hsa-mir-197 | hsa_circ_0001610 (up) | Breast cancer | up | Potential blood-based biomarker for breast cancer screening. | [60] |
| hsa-mir-503 | hsa_circ_0004365 (up) | Breast cancer | up | Inhibits cell proliferation by suppressing CCND1 expression. | [61] |
| hsa-mir-503 | hsa_circ_0004365 (up) | Gastric cancer | down | Regulates cisplatin resistance by targeting IGF1R and BCL2. | [62] |
| hsa-mir-503 | hsa_circ_0004365 (up) | Laryngeal carcinoma | down | Enhances cell radiosensitivity via inhibition of WEE1. | [63] |
| hsa-mir-503 | hsa_circ_0004365 (up) | Ovarian cancer | up | Associated with chemoresistance and tumor progression. | [64] |
| hsa-mir-503-3p |  | Breast cancer | up | Promotes EMT by directly targeting SMAD2 and E-cadherin. | [65] |
| hsa-mir-942 | hsa_circ_0006411 (down) | Esophageal squamous cell carcinoma | up | Promotes cancer stem cell-like traits through activation of Wnt/β-catenin signalling pathway. | [66] |

EMT: Epithelial-to-mesenchymal-transition

**Supporting information**

**Table S1.** Primer sequences for qPCR

**Table S2.** Primary antibodies used for western blotting and immunocytochemistry

**Table S3.** Growth parameters of FIR20 and control cell lines.

**Table S4.** Radiation sensitivity parameters of cell lines.

**Table S5.** FIR20 enriched genes that overlap with Post et al and Weichselbaum et al genes.

**Table S6:** 7 high-confidence DECs and annotations.

**Table S7:** Published function of DECs.

**Table S8:** Published function and clinical significance of some miRNA targets of the DECs.

**Fig. S1.** Cell cycle analysis

**Fig. S2.** Gene set enrichment analysis of FIR20 transcriptome

**Fig. S3**. Tamoxifen cytotoxicity is attenuated in FIR20 cells

**Fig. S4.** Enrichment analysis of the 108 predicted target genes of the dysregulated DECs.

**Fig. S5.** Functional enrichment analysis of all (8287) miRWalk predicted target genes.

Data S1_DEMs.xlsx

Data S2_Core signature_Enrichment analysis.xlsx

Data S3_Extended siganture_Transcription factor analysis.xlsx

Data S4_FIR20vsREST transcriptome_GSEA.xlsx

Data S5_Patient data analysis.xlsx

Data S6_DECs.xlsx

Data S7A_ceRNA Network default node.xlsx

Data S7B_ceRNA network.cys

Data S8_CircRNAup_mRNAup_Enrichment_PPI network.xlsx

**References**

1. Shannon, P., Markiel, A., Ozier, O., Baliga, N. S., Wang, J. T., Ramage, D., … Ideker, T. (2003). Cytoscape: A software Environment for integrated models of biomolecular interaction networks. *Genome Research*, *13*(11), 2498–2504. https://doi.org/10.1101/gr.1239303

2. Kuleshov, M. V., Jones, M. R., Rouillard, A. D., Fernandez, N. F., Duan, Q., Wang, Z., … Ma’ayan, A. (2016). Enrichr: a comprehensive gene set enrichment analysis web server 2016 update. *Nucleic acids research*, *44*(W1), W90–W97. https://doi.org/10.1093/nar/gkw377

3. Li, Y., Zheng, Q., Bao, C., Li, S., Guo, W., Zhao, J., … Huang, S. (2015). Circular RNA is enriched and stable in exosomes: A promising biomarker for cancer diagnosis. *Cell Research*, *25*(8), 981–984. https://doi.org/10.1038/cr.2015.82

4. Post, A. E. M., Smid, M., Nagelkerke, A., Martens, J. W. M., Bussink, J., Sweep, F. C. G. J., & Span, P. N. (2018). Interferon-stimulated genes are involved in cross-resistance to radiotherapy in tamoxifen-resistant breast cancer. *Clinical Cancer Research*, *24*(14), 3397–3408. https://doi.org/10.1158/1078-0432.CCR-17-2551

5. Weichselbaum, R. R., Ishwaran, H., Yoon, T., Nuyten, D. S. A., Baker, S. W., Khodarev, N., … Minn, A. J. (2008). An interferon-related gene signature for DNA damage resistance is a predictive marker for chemotherapy and radiation for breast cancer. *Proceedings of the National Academy of Sciences of the United States of America*, *105*(47), 18490–18495. Retrieved from /pmc/articles/PMC2587578/

6. Jeck, W. R., Sorrentino, J. A., Wang, K., Slevin, M. K., Burd, C. E., Liu, J., … Sharpless, N. E. (2013). Circular RNAs are abundant, conserved, and associated with ALU repeats. *Rna*, *19*(2), 141–157. https://doi.org/10.1261/rna.035667.112

7. PG, M., P, G., S, M., G, D., I, H., L, S., … N, R. (2017). A map of human circular RNAs in clinically relevant tissues. *Journal of molecular medicine (Berlin, Germany)*, *95*(11), 1179–1189. https://doi.org/10.1007/S00109-017-1582-9

8. Rybak-Wolf, A., Stottmeister, C., Glažar, P., Jens, M., Pino, N., Hanan, M., … Rajewsky, N. (2014). Circular RNAs in the Mammalian Brain Are Highly Abundant, Conserved, and Dynamically Expressed. *Molecular Cell*, *58*(5), 870–885. https://doi.org/10.1016/j.molcel.2015.03.027

9. Salzman, J., Chen, R. E., Olsen, M. N., Wang, P. L., & Brown, P. O. (2013). Cell-Type Specific Features of Circular RNA Expression. *PLoS Genetics*, *9*(9), e1003777. https://doi.org/10.1371/journal.pgen.1003777

10. Memczak, S., Jens, M., Elefsinioti, A., Torti, F., Krueger, J., Rybak, A., … Rajewsky, N. (2013). Circular RNAs are a large class of animal RNAs with regulatory potency. *Nature*, *495*(7441), 333–338. https://doi.org/10.1038/nature11928

11. Du, G., Ma, R., Li, H., He, J., Feng, K., Niu, D., & Yin, D. (2021). Increased Expression of hsa_circ_0002111 and Its Clinical Significance in Papillary Thyroid Cancer. *Frontiers in Oncology*, *11*, 258. https://doi.org/10.3389/fonc.2021.644011

12. Peng, N., Shi, L., Zhang, Q., Hu, Y., Wang, N., & Ye, H. (2017). Microarray profiling of circular RNAs in human papillary thyroid carcinoma. *PLoS ONE*, *12*(3). https://doi.org/10.1371/journal.pone.0170287

13. Guo, D., Li, F., Zhao, X., Long, B., Zhang, S., Wang, A., … Li, B. (2020). Circular RNA expression and association with the clinicopathological characteristics in papillary thyroid carcinoma. *Oncology Reports*, *44*(2), 519–532. https://doi.org/10.3892/or.2020.7626

14. Lan, X., Xu, J., Chen, C., Zheng, C., Wang, J., Cao, J., … Ge, M. (2018). The landscape of circular RNA expression profiles in papillary thyroid carcinoma based on RNA sequencing. *Cellular Physiology and Biochemistry*, *47*(3), 1122–1132. https://doi.org/10.1159/000490188

15. Xie, Y., Shao, Y., Sun, W., Ye, G., Zhang, X., Xiao, B., & Guo, J. (2018). Downregulated expression of hsa-circ-0074362 in gastric cancer and its potential diagnostic values. *Biomarkers in Medicine*, *12*(1), 11–20. https://doi.org/10.2217/bmm-2017-0114

16. Liu, Y., Chang, Y., & Cai, Y. (2020). circTNFRSF21, a newly identified circular RNA promotes endometrial carcinoma pathogenesis through regulating miR-1227-MAPK13/ATF2 axis. *Aging*, *12*(8), 6774–6792. https://doi.org/10.18632/aging.103037

17. Lin, J., Zhang, Y., Zeng, X., Xue, C., & Lin, X. (2020). CircRNA circRIMS acts as a microRNA sponge to promote gastric cancer metastasis. *ACS Omega*, *5*(36), 23237–23246. https://doi.org/10.1021/acsomega.0c02991

18. Zhu, J., Ye, J., Zhang, L., Xia, L., Hu, H., Jiang, H., … Luo, C. (2017). Differential Expression of Circular RNAs in Glioblastoma Multiforme and Its Correlation with Prognosis. *Translational Oncology*, *10*(2), 271–279. https://doi.org/10.1016/j.tranon.2016.12.006

19. Xu, J., Zhang, Y., Huang, Y., Dong, X., Xiang, Z., Zou, J., … Lu, W. (2020). circEYA1 Functions as a Sponge of miR-582-3p to Suppress Cervical Adenocarcinoma Tumorigenesis via Upregulating CXCL14. *Molecular Therapy - Nucleic Acids*, *22*, 1176–1190. https://doi.org/10.1016/j.omtn.2020.10.026

20. van den Berg, I., Smid, M., Coebergh van den Braak, R. R. J., van Deurzen, C. H. M., de Weerd, V., Foekens, J. A., … Wilting, S. M. (2021). Circular rna in chemonaive lymph node negative colon cancer patients. *Cancers*, *13*(8). https://doi.org/10.3390/cancers13081903

21. Png, K. J., Yoshida, M., Zhang, X. H. F., Shu, W., Lee, H., Rimner, A., … Tavazoie, S. F. (2011). MicroRNA-335 inhibits tumor reinitiation and is silenced through genetic and epigenetic mechanisms in human breast cancer. *Genes and Development*, *25*(3), 226–231. https://doi.org/10.1101/gad.1974211

22. Guo, X., Yu, L., Zhang, Z., Dai, G., Gao, T., & Guo, W. (2017). miR-335 negatively regulates osteosarcoma stem cell-like properties by targeting POU5F1. *Cancer Cell International*, *17*(1), 29. https://doi.org/10.1186/s12935-017-0398-6

23. Cheng, Y., & Shen, P. (2020). miR-335 Acts as a Tumor Suppressor and Enhances Ionizing Radiation-Induced Tumor Regression by Targeting ROCK1. *Frontiers in oncology*, *10*, 278. https://doi.org/10.3389/fonc.2020.00278

24. Luo, Y., Tong, L., Meng, H., Zhu, W., Guo, L., Wei, T., & Zhang, J. (2017). MiR-335 regulates the chemo-radioresistance of small cell lung cancer cells by targeting PARP-1. *Gene*, *600*, 9–15. https://doi.org/10.1016/j.gene.2016.11.031

25. Yang, N., & Zhang, D. (2019). MiR-335-5p inhibits cell proliferation, migration and invasion in colorectal cancer through downregulating LDHB. *JBUON*, *24*(3), 1128–1136.

26. Zhang, L. L., Zhang, L. F., Guo, X. H., Zhang, D. Z., Yang, F., & Fan, Y. Y. (2018). Downregulation of miR-335-5p by Long Noncoding RNA ZEB1-AS1 in Gastric Cancer Promotes Tumor Proliferation and Invasion. *DNA and Cell Biology*, *37*(1), 46–52. https://doi.org/10.1089/dna.2017.3926

27. Dong, Y., Liu, Y., Jiang, A., Li, R., Yin, M., & Wang, Y. (2018). MicroRNA-335 suppresses the proliferation, migration, and invasion of breast cancer cells by targeting EphA4. *Molecular and Cellular Biochemistry*, *439*(1–2), 95–104. https://doi.org/10.1007/s11010-017-3139-1

28. Gao, Y., Zeng, F., Wu, J. Y., Li, H. Y., Fan, J. J., Mai, L., … Song, F. zhou. (2015). MiR-335 inhibits migration of breast cancer cells through targeting oncoprotein c-Met. *Tumor Biology*, *36*(4), 2875–2883. https://doi.org/10.1007/s13277-014-2917-6

29. Hao, J., Lai, M., & Liu, C. (2019). Expression of MIR-335 in triple-negative breast cancer and its effect on chemosensitivity. *Journal of B.U.ON.*, *24*(4), 1526–1531. Retrieved from https://europepmc.org/article/med/31646803

30. Heyn, H., Engelmann, M., Schreek, S., Ahrens, P., Lehmann, U., Kreipe, H., … Beger, C. (2011). MicroRNA miR-335 is crucial for the BRCA1 regulatory cascade in breast cancer development. *International Journal of Cancer*, *129*(12), 2797–2806. https://doi.org/10.1002/ijc.25962

31. Chen, J.-H. H., Huang, W.-C. C., Bamodu, O. A., Chang, P. M. H., Chao, T.-Y. Y., Huang, T.-H. H., … Huang, T.-H. H. (2019). Monospecific antibody targeting of CDH11 inhibits epithelial-to-mesenchymal transition and represses cancer stem cell-like phenotype by up-regulating miR-335 in metastatic breast cancer, in vitro and in vivo. *BMC Cancer*, *19*(1). https://doi.org/10.1186/s12885-019-5811-1

32. Jia, Q., Ye, L., Xu, S., Xiao, H., Xu, S., Shi, Z., … Chen, Z. (2020). Circular RNA 0007255 regulates the progression of breast cancer through miR-335-5p/SIX2 axis. *Thoracic Cancer*, *11*(3), 619–630. https://doi.org/10.1111/1759-7714.13306

33. Gao, Y., Wang, Y., Wang, X., Zhao, C., Wang, F., Du, J., … Zhang, J. (2021). miR-335-5p suppresses gastric cancer progression by targeting MAPK10. *Cancer Cell Int*, *21*, 71. https://doi.org/10.1186/s12935-020-01684-z

34. Sun, X., Lin, F., Sun, W., Zhu, W., Fang, D., Luo, L., … Jiang, L. (2021). Exosome-transmitted miRNA-335-5p promotes colorectal cancer invasion and metastasis by facilitating EMT via targeting RASA1. *Molecular Therapy - Nucleic Acids*, *24*, 164–174. https://doi.org/10.1016/j.omtn.2021.02.022

35. Chen, D., Bao, C., Zhao, F., Yu, H., Zhong, G., Xu, L., & Yan, S. (2020). Exploring Specific miRNA-mRNA Axes With Relationship to Taxanes-Resistance in Breast Cancer. *Frontiers in Oncology*, *10*, 1397. https://doi.org/10.3389/fonc.2020.01397

36. Cao, J., Cai, J., Huang, D., Han, Q., Chen, Y., Yang, Q., … Wang, Z. (2014). miR-335 Represents an Independent Prognostic Marker in Epithelial Ovarian Cancer. *Am J Clin Pathol*, *141*, 437. https://doi.org/10.1309/AJCPLYTZGB54ISZC

37. Martin, E. C., Conger, A. K., Yan, T. J., Hoang, V. T., Miller, D. F. B., Buechlein, A., … Burow, M. E. (2017). MicroRNA-335-5p and -3p synergize to inhibit estrogen receptor alpha expression and promote tamoxifen resistance. *FEBS Letters*, *591*(2), 382–392. https://doi.org/10.1002/1873-3468.12538

38. Shen, Y., Ye, Y. F., Ruan, L. W., Bao, L., Wu, M. W., & Zhou, Y. (2017). Inhibition of miR-660-5p expression suppresses tumor development and metastasis in human breast cancer. *Genetics and Molecular Research*, *16*(1). https://doi.org/10.4238/gmr16019479

39. Sun, L. L., Wang, J., Zhao, Z. J., Liu, N., Wang, A. L., Ren, H. Y., … Mi, X. Y. (2014). Suppressive role of miR-502-5p in breast cancer via downregulation of TRAF2. *Oncology Reports*, *31*(5), 2085–2092. https://doi.org/10.3892/or.2014.3105

40. Zhang, H. Da, Sun, D. W., Mao, L., Zhang, J., Jiang, L. H., Li, J., … Tang, J. H. (2015). MiR-139-5p inhibits the biological function of breast cancer cells by targeting Notch1 and mediates chemosensitivity to docetaxel. *Biochemical and Biophysical Research Communications*, *465*(4), 702–713. https://doi.org/10.1016/j.bbrc.2015.08.053

41. Xu, K., Shen, K., Liang, X., Li, Y., Nagao, N., Li, J., … Yin, P. (2016). MiR-139-5p reverses CD44+/CD133+-associated multidrug resistance by downregulating NOTCH1 in colorectal carcinoma cells. *Oncotarget*, *7*(46), 75118–75129. https://doi.org/10.18632/oncotarget.12611

42. Shao, Q., Zhang, P., Ma, Y., Lu, Z., Meng, J., Li, H., … Ma, S. (2018). MicroRNA-139-5p affects cisplatin sensitivity in human nasopharyngeal carcinoma cells by regulating the epithelial-to-mesenchymal transition. *Gene*, *652*, 48–58. https://doi.org/10.1016/j.gene.2018.02.003

43. Mesci, A., Huang, X., Taeb, S., Jahangiri, S., Kim, Y., Fokas, E., … Liu, S. K. (2017). Targeting of CCBE1 by miR-330-3p in human breast cancer promotes metastasis. *British Journal of Cancer*, *116*(10), 1350–1357. https://doi.org/10.1038/bjc.2017.105

44. Wang, H., Chen, S. H., Kong, P., Zhang, L. Y., Zhang, L. L., Zhang, N. Q., & Gu, H. (2018). Increased expression of miR-330-3p: a novel independent indicator of poor prognosis in human breast cancer. *European review for medical and pharmacological sciences*, *22*(6), 1726–1730. https://doi.org/10.26355/eurrev_201803_14587

45. Yang, F., Luo, L. ji, Zhang, L., Wang, D. dan, Yang, S. jin, Ding, L., … Tang, J. hai. (2017). MiR-346 promotes the biological function of breast cancer cells by targeting SRCIN1 and reduces chemosensitivity to docetaxel. *Gene*, *600*, 21–28. https://doi.org/10.1016/j.gene.2016.11.037

46. Guo, Z., Li, J., Sun, J., Sun, L., Zhou, Y., & Yu, Z. (2018). MiR-346 promotes HCC progression by suppressing breast cancer metastasis suppressor 1 expression. *Oncology Research*, *26*(7), 1073–1081. https://doi.org/10.3727/096504017X15145088802439

47. Bozec, A., Zangari, J., Butori-Pepino, M., Ilie, M., Lalvee, S., Juhel, T., … Vouret-Craviari, V. (2017). MiR-223-3p inhibits angiogenesis and promotes resistance to cetuximab in head and neck squamous cell carcinoma. *Oncotarget*, *8*(34), 57174–57186. https://doi.org/10.18632/oncotarget.19170

48. Sun, S., Wang, X., Xu, X., Di, H., Du, J., Xu, B., … Wang, J. (2017). MiR-433-3p suppresses cell growth and enhances chemosensitivity by targeting CREB in human glioma. *Oncotarget*, *8*(3), 5057–5068. https://doi.org/10.18632/oncotarget.13789

49. Lin, W. C., Chen, L. H., Hsieh, Y. C., Yang, P. W., Lai, L. C., Chuang, E. Y., … Tsai, M. H. (2019). MiR-338-5p inhibits cell proliferation, colony formation, migration and cisplatin resistance in esophageal squamous cancer cells by targeting FERMT2. *Carcinogenesis*, *40*(7), 883–892. https://doi.org/10.1093/carcin/bgy189

50. Ren, G. B., Wang, L., Zhang, F. H., Meng, X. R., & Mao, Z. P. (2016). Study on the relationship between miR-520g and the development of breast cancer. *European Review for Medical and Pharmacological Sciences*, *20*(4), 657–663.

51. Peng, C., Huang, K., Liu, G., Li, Y., & Yu, C. (2019). MiR-876-3p regulates cisplatin resistance and stem cell-like properties of gastric cancer cells by targeting TMED3. *Journal of Gastroenterology and Hepatology (Australia)*, *34*(10), 1711–1719. https://doi.org/10.1111/jgh.14649

52. Zhang, L., Sullivan, P. S., Goodman, J. C., Gunaratne, P. H., & Marchetti, D. (2011). MicroRNA-1258 suppresses breast cancer brain metastasis by targeting heparanase. *Cancer Research*, *71*(3), 645–654. https://doi.org/10.1158/0008-5472.CAN-10-1910

53. Cheng, Y., Xiang, G., Meng, Y., & Dong, R. (2016). MiRNA-183-5p promotes cell proliferation and inhibits apoptosis in human breast cancer by targeting the PDCD4. *Reproductive Biology*, *16*(3), 225–233. https://doi.org/10.1016/j.repbio.2016.07.002

54. Zhao, L., Gu, H., Chang, J., Wu, J., Wang, D., Chen, S., … Qian, B. (2014). MicroRNA-383 regulates the apoptosis of tumor cells through targeting Gadd45g. *PLoS ONE*, *9*(11). https://doi.org/10.1371/journal.pone.0110472

55. Jiang, J., Xie, C., Liu, Y., Shi, Q., & Chen, Y. (2019). Up-regulation of miR-383-5p suppresses proliferation and enhances chemosensitivity in ovarian cancer cells by targeting TRIM27. *Biomedicine and Pharmacotherapy*, *109*, 595–601. https://doi.org/10.1016/j.biopha.2018.10.148

56. Majumder, M., Landman, E., Liu, L., Hess, D., & Lala, P. K. (2015). COX-2 elevates oncogenic miR-526b in breast cancer by EP4 activation. *Molecular Cancer Research*, *13*(6), 1022–1033. https://doi.org/10.1158/1541-7786.MCR-14-0543

57. Shin, B., Feser, R., Nault, B., Hunter, S., Maiti, S., Ugwuagbo, K. C., & Majumder, M. (2019). miR526b and miR655 induce oxidative stress in breast cancer. *International Journal of Molecular Sciences*, *20*(16). https://doi.org/10.3390/ijms20164039

58. Dai, N., Qing, Y., Cun, Y., Zhong, Z., Li, C., Zhang, S., … Wang, D. (2018). miR-513a-5p regulates radiosensitivity of osteosarcoma by targeting human apurinic/apyrimidinic endonuclease. *Oncotarget*, *9*(39), 25414–25426. https://doi.org/10.18632/oncotarget.11003

59. Sun, W. juan, Zhang, Y. na, & Xue, P. (2019). miR-186 inhibits proliferation, migration, and epithelial-mesenchymal transition in breast cancer cells by targeting Twist1. *Journal of Cellular Biochemistry*, *120*(6), 10001–10009. https://doi.org/10.1002/jcb.28283

60. Shaker, O., Maher, M., Nassar, Y., Morcos, G., & Gad, Z. (2015). Role of microRNAs -29b-2, -155, -197 and -205 as diagnostic biomarkers in serum of breast cancer females. *Gene*, *560*(1), 77–82. https://doi.org/10.1016/j.gene.2015.01.062

61. Long, J., Ou, C., Xia, H., Zhu, Y., & Liu, D. (2015). MiR-503 inhibited cell proliferation of human breast cancer cells by suppressing CCND1 expression. *Tumor Biology*, *36*(11), 8697–8702. https://doi.org/10.1007/s13277-015-3623-8

62. Wang, T. S., Ge, G. X., Ding, Y., Zhou, X., Huang, Z. B., Zhu, W., … Liu, P. (2014). MiR-503 regulates cisplatin resistance of human gastric cancer cell lines by targeting IGF1R and BCL2. *Chinese Medical Journal*, *127*(12), 2357–2362. https://doi.org/10.3760/cma.j.issn.0366-6999.20140318

63. Ma, H., Lian, R., Wu, Z., Li, X., Yu, W., Shang, Y., & Guo, X. (2017). MiR-503 enhances the radiosensitivity of laryngeal carcinoma cells via the inhibition of WEE1. *Tumor Biology*, *39*(10), 1–9. https://doi.org/10.1177/1010428317706224

64. Park, Y. T., Jeong, J. Y., Lee, M. J., Kim, K. Il, Kim, T. H., Kwon, Y. Do, … An, H. J. (2013). MicroRNAs overexpressed in ovarian ALDH1-positive cells are associated with chemoresistance. *Journal of Ovarian Research*, *6*(1). https://doi.org/10.1186/1757-2215-6-18

65. Zhao, Z., Fan, X., Jiang, L., Xu, Z., Xue, L., Zhan, Q., & Song, Y. (2017). miR-503-3p promotes epithelial–mesenchymal transition in breast cancer by directly targeting SMAD2 and E-cadherin. *Journal of Genetics and Genomics*, *44*(2), 75–84. https://doi.org/10.1016/j.jgg.2016.10.005

66. Ge, C., Wu, S., Wang, W., Liu, Z., Zhang, J., Wang, Z. Z., … Song, X. (2015). miR-942 promotes cancer stem cell-like traits in esophageal squamous cell carcinoma through activation of Wnt/β-catenin signalling pathway. *Oncotarget*, *6*(13), 10964–10977. https://doi.org/10.18632/oncotarget.3696
